# Hydrogen vs. Halogen Bonds in 1-Halo-*Closo*-Carboranes

**DOI:** 10.3390/ma13092163

**Published:** 2020-05-07

**Authors:** Ibon Alkorta, Jose Elguero, Josep M. Oliva-Enrich

**Affiliations:** 1 Instituto de Química Médica, CSIC, Juan de la Cierva, 3, E-28006 Madrid, Spain; iqmbe17@iqm.csic.es; 2 Instituto de Química-Física “Rocasolano”, CSIC, Serrano, 119, E-28006 Madrid, Spain; j.m.oliva@iqfr.csic.es

**Keywords:** *closo*-carboranes, hydrogen bonds, halogen bonds, 1-halobenzenes

## Abstract

A theoretical study of the hydrogen bond (HB) and halogen bond (XB) complexes between 1-halo-*closo*-carboranes and hydrogen cyanide (NCH) as HB and XB probe has been carried out at the MP2 computational level. The energy results show that the HB complexes are more stable than the XBs for the same system, with the exception of the isoenergetic iodine derivatives. The analysis of the electron density with the quantum theory of atoms in molecules (QTAIM) shows the presence of a unique intermolecular bond critical point with the typical features of weak noncovalent interactions (small values of the electron density and positive Laplacian and total energy density). The natural energy decomposition analysis (NEDA) of the complexes shows that the HB and XB complexes are dominated by the charge-transfer and polarization terms, respectively. The work has been complemented with a search in the CSD database of analogous complexes and the comparison of the results, with those of the 1-halobenzene:NCH complexes showing smaller binding energies and larger intermolecular distances as compared to the 1-halo-*closo*-carboranes:NCH complexes.

## 1. Introduction

The most important noncovalent interaction is, without doubt, the hydrogen bond. However, in recent years other interactions [1] have been described in various studies. The study of the halogen bond has taken place in stages, with the initial experimental and theoretical studies being carried out in the middle of the 20th century [2]. In the last years of the 20th century, systematic studies of the gas phase and its comparison with analogous hydrogen bonds were carried out, mainly by Legon and co-workers [3]. In this century, the properties of the halogen bond have found applications in the fields of drug design [4,5,6], material science [7,8,9] and organocatalysis [10,11,12].

The halogen bond has been computationally characterized by the interaction of a region of positive electrostatic potential on the halogen atoms, known as the σ-hole, with the negative electrostatic potential of a Lewis base [13,14,15,16]. A large number of different Lewis bases have been used to form such complexes [11], including carbenes [17,18], boron derivatives [19,20,21,22], σ-bonds [23,24] and π-systems [25,26]. As with the hydrogen bond, the effects of cooperativity [27,28] in the clusters formed by halogen bonds or in connection with other noncovalent bonds have been observed [29,30,31,32,33]. Furthermore, the limits of the halogen bonds have been expanded to encompass shared and ionic bonds [34,35,36,37,38,39]. Studies on energy decomposition analysis suggest that charge-transfer is the dominant term [40,41,42,43,44], while in other cases the electrostatic and dispersion terms were found to be more important [45,46,47,48].

**Scheme 1:**
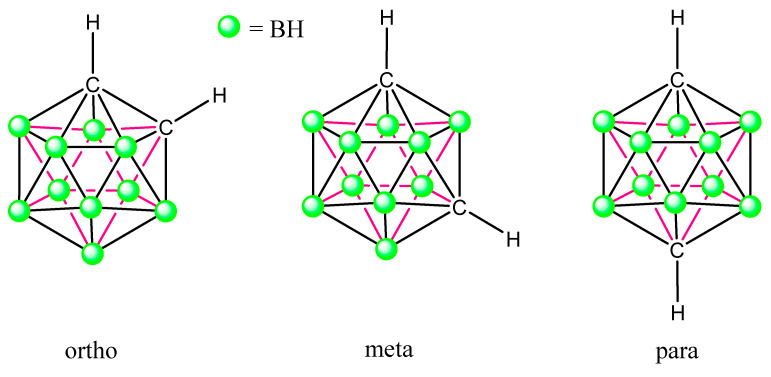
Parent *ortho*, *meta* and *para closo*-carboranes.

*Closo*-carboranes are polyhedral compounds formed by boron, carbon and hydrogen with non-open vertices; the 12-vertex icosahedral cage is the most commonly studied [49]. The parent compound (closo-B_10_H_12_C_2_) has three different isomers depending on the relative disposition of the carbon atoms: *ortho (closo*-1,2-dicarbadodecarborane), *meta (closo*-1,7-dicarbadodecarborane) and *para* (*closo*-1,12-dicarbadodecarborane) (Scheme 1). Theoretical studies of the interaction of carboranes with biomolecules by means of dihydrogen bonds [50], the hydrogen bond with π-systems [51,52], halogen bonds [53,54], interaction with gold derivatives [55,56] and as superacids [57] have been reported.

**Scheme 2:**
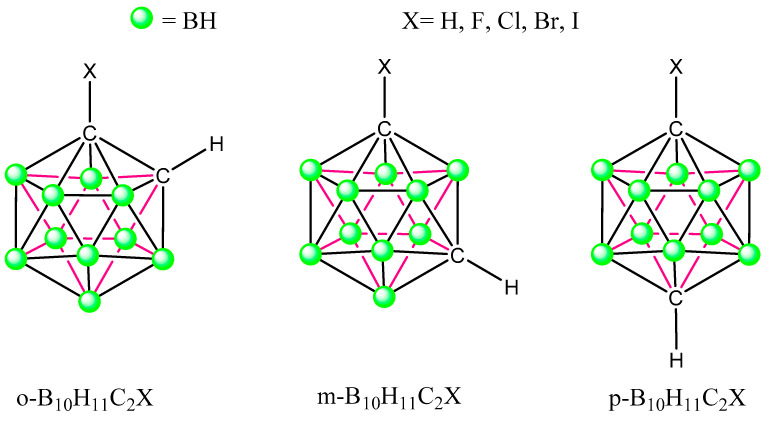
The 1-halo-*closo*-carboranes under consideration in this article.

In this article, we studied the complexes between a Lewis base, hydrogen cyanide (NCH), used as a hydrogen bond (HB) and halogen bond (XB) acceptor probe and 1-halo-*closo*-carboranes (Scheme 2) acting as hydrogen- and halogen-bond donors. The parent compounds X = H have also been considered. In addition, a search in the CSD database has been carried out in order to identify systems with similar interactions to those studied here. Finally, the analogous complexes with 1-halobenzene derivatives have been included and compared with those of the 1-halo-*closo*-carboranes.

## 2. Computational Methods

The geometry of the isolated monomers and complexes was optimized at the MP2 computational level [58] with the aug-cc-pVDZ basis set [59] for all atoms except for iodine, where the effective core potential basis set, aug-cc-pVDZ-pp [60], was used. Frequency calculations at the same computational level were carried out to confirm that the structures are energy minima. In order to have more reliable basis sets, the complete basis set (CBS) extrapolation was calculated using MP2/aug-cc-pVDZ/aug-cc-pVDZ-pp and MP2/aug-cc-pVTZ/aug-cc-pVTZ-pp energies and Equations (1)–(3) [61,62]:

(1) $$E_{n}^{HF}=E_{CBS}^{HF}+Ae^{-n\alpha }$$

(2) $$E_{n}^{MP2-corr}=E_{CBS}^{MP2-corr}+Bn^{-3}$$

(3) $$E_{CBS}^{MP2}=E_{CBS}^{HF}+E_{CBS}^{MP2-corr}$$

where n is 2 or 3 for the energetic values obtained with the aug-cc-pVDZ/aug-cc-pVDZ-pp and aug-cc-pVTZ/aug-cc-pVTZ-pp, respectively, and α = 1.43 [61]. All these calculations were carried out using the Gaussian 16 and the Molpro packages [63,64].

The dissociation energy was calculated as the difference between the sum of the CBS energies of the monomers in their minima configuration and the energy of the complex, Equation (4).

De = E(A) + E(B) − E(AB)(4)

The molecular electrostatic potential of the isolated monomers was calculated with the Gaussian 16 program. The local maxima on the 0.001 au electron density isosurface, V_s,max_, also known as the σ-hole [65,66], were located with the Multiwfn program [67] and represented with the JMol program [68].

The electron density of the systems was analyzed within the quantum theory of atoms in molecules (QTAIM) [69,70]. In this methodology, the critical points found between two atoms, called bond critical points (BCP), provide information about the nature of such bonds. In the case of intermolecular BCP, the values of the electron density (ρ_BCP_), Laplacian (▽^2^ρ_BCP_) and total energy density (H_BCP_) indicate the closed/open shell and covalent nature of the interaction [71,72]. The QTAIM calculations were carried out with the AIMAll program [73]. In addition, the noncovalent index (NCI) [74], which is based on the reduced gradient density (RGD) and the sign of the second eigenvalue of the curvature, λ_2_, was calculated with the NCIPLOT [75] and Multiwfn [67] programs and represented by the VMD program [76].

The natural energy decomposition analysis (NEDA) [77,78] method, which utilizes the natural bond orbitals [79], was used to obtain information on the importance of the different energy terms in the interaction. This methodology divides the interaction energy into the four attractive components: (i) orbital charge-transfer that arises from the electron delocalization from one monomer to the other, (ii) classical electrostatic interaction of the monomers, (iii) polarization and (iv) exchange-correlation term. Finally, the repulsive components take into account the electronic deformation due to the complex formation in each monomer. These calculations were performed at the PBE0/aug-cc-pVDZ computational level with the NBO-7 program [80].

A search was carried out in the Cambridge Structural Database (CSD) [81] version 5.41, updated in November 2019 and March 2020, for structures with *closo*-carborane molecules showing hydrogen and halogen bonds. The structures found in this study were analyzed and compared to the computational results.

## 3. Results and Discussions

### 3.1. Isolated 1-Halo-Closo-Carboranes

The optimized geometry of the isolated 1-halo-*closo*-carboranes (Table S1) maintained the icosahedral geometry of the parent compounds with minimal distortion of the geometry. Only a small elongation (<0.01 Å) of the bonds surrounding the C-X group was observed. Based on the NBO analysis, this effect was due to the charge-transfer of the halogen lone pair orbitals to the C-C bond in the *ortho* derivatives and C-B bond in the *meta* and *para* derivatives.

**Figure 1:**
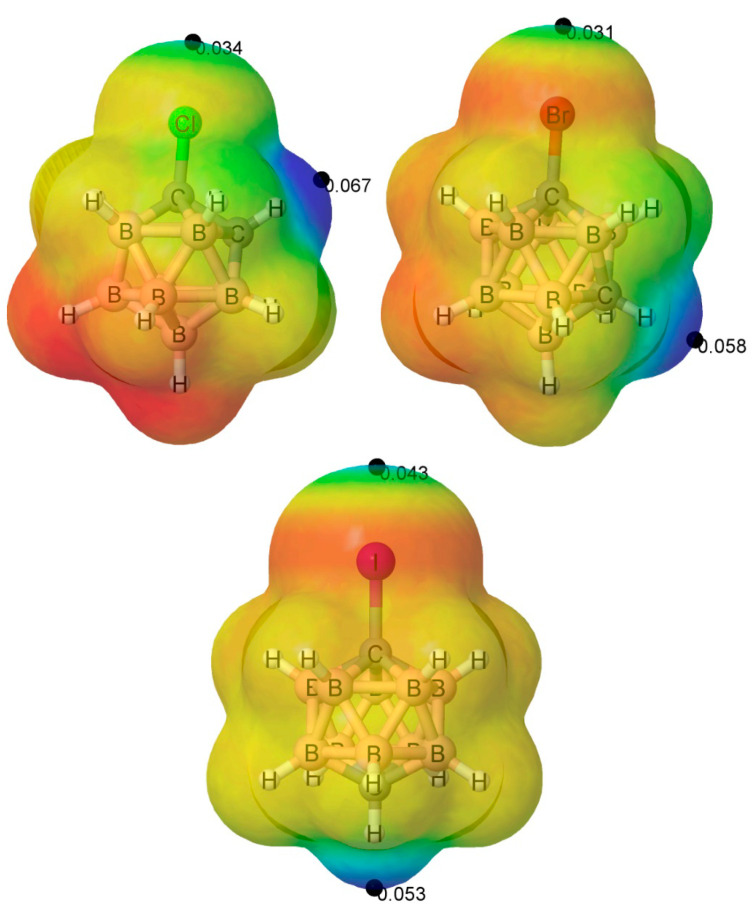
The molecular electrostatic potential (MESP) of three of the systems studied. The MESP energy values range between −0.015 (red) and 0.05 au (blue). The location of the V_s,max_ is indicated with black circles, and its value is indicated in au.

The molecular electrostatic potential on the 0.001 au electron density isosurface (MESP) provided clues to the potential positions of hydrogen- and halogen-bond donors as well as their relative binding energy. In all cases, local maxima on the MESP, V_s,max_, with positive values were located close to the positions of the atoms bonded to the carbon atoms, H and X, except those surrounding the fluorine atoms where negative values were found (Table 1). Figure 1 shows three examples of the calculated MESP with the location and value of the V_s,max_.

The V_s,max_ values associated with the H atoms were smaller in the parent compounds than in the 1-halo derivatives, except for the *ortho* derivative, but no significant differences were found between the different halogen derivatives in a given isomer. Regarding the isomers, the largest values were observed in the *ortho* derivatives, followed by the *meta*, with the *para* having the smallest values. The V_s,max_ value increased in a given isomer with the size of the halogen atom, in agreement with previous studies [16]. When the different isomers were compared for a given halogen atom, the same trend as previously mentioned for the H values was observed: *ortho* > *meta* > *para*.

### 3.2. 1-Halo-Closo-Carboranes in Hydrogen and Halogen Bonds

**Figure 2:**
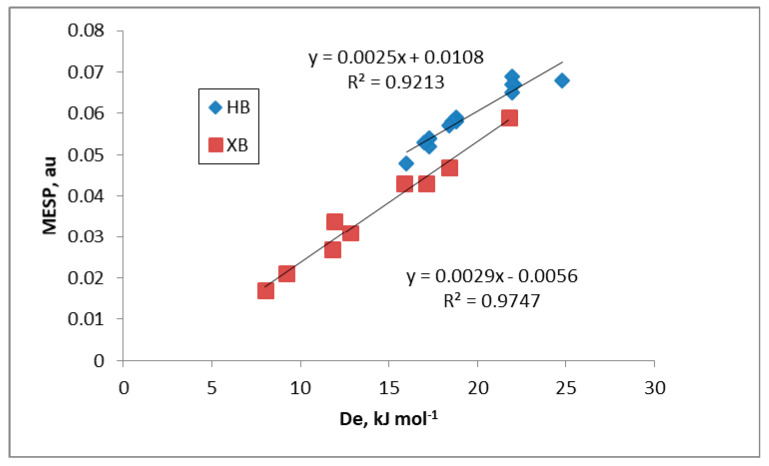
The MESP (au) vs. De (kJ·mol^−1^) in the hydrogen bond (HB) and halogen bond (XB) complexes.

The energy minima structures (Table S2) were obtained for all the 1-halo-*closo*-carboranes acting as HB and XB donors vs. NCH, except for the 1-fluoro derivatives where no minima have been found when acting as expected as an XB donor, based on the MESP values previously discussed. The dissociation energy values are shown in Table 2. The largest values for a given substituent and type of interaction (HB or XB) were found in the *ortho* isomer complexes, followed by the *meta* isomer, with the *para* derivatives being the weakest. These results are in agreement with the MESP values reported in Table 1. In fact, the representation of the dissociation energies vs. the MESP V_s,max_ of the corresponding isolated monomer and atom involved in the interaction provides linear correlations for both HB and XB interactions (Figure 2). The fact that the HB complex of the *o*-B_10_H_12_C_2_ corresponded to the only parent compound with larger De than the halogenated derivatives in the corresponding series, with this also being the worst-fitted point in Figure 2, will be discussed later.

The XB complexes of the chlorine and bromine derivatives were less stable than the corresponding HB ones, while the XB and HB showed similar energies for the iodine derivative. The XBs formed by the chlorine derivatives were the least stable in each series, increasing on average by 3.8 kJ·mol^−1^ when replaced by bromine; a further increment of 5.6 kJ·mol^−1^ was obtained when the halogen was iodine.

**Figure 3:**
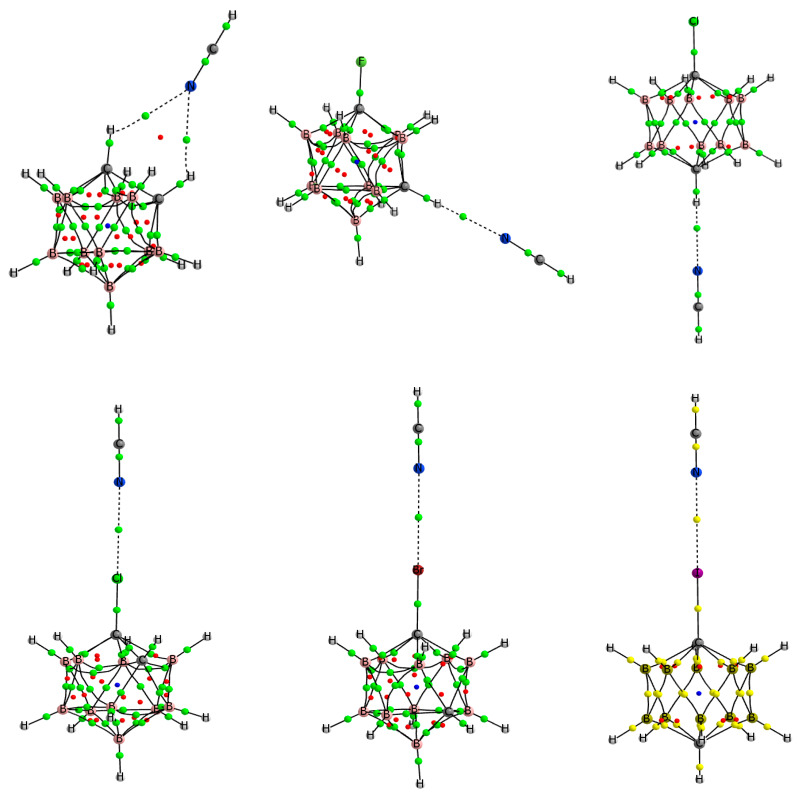
Molecular graph of some of the hydrogen- and halogen-bonded complexes between 1-halo-*closo*-carboranes and NCH. The locations of the bond, ring and cage critical points are indicated with green, red and blue dots, respectively.

**Figure 4:**
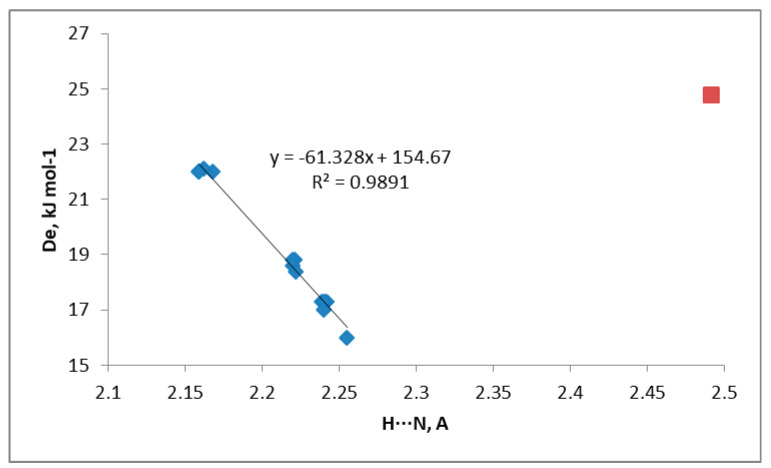
Intermolecular H⋯N distances, Å, vs. the dissociation energy, kJ·mol^−1^, in the HB complexes. The *o*-B_10_H_12_C_2_:NCH complex is indicated with a red square, and it is not included in the correlation equation.

The intermolecular distances of the complexes are shown in Table 3, and some of their geometries have been represented in Figure 3. The first interesting fact is that in all complexes, the interaction involved one atom from each molecule, except for the case of the *o*-B_10_H_12_C_2_:NCH (HB) complex where the two CH groups in *ortho* were interacting with the N atom of the NCH molecule in a bifurcated HB [82,83]. The features of this geometry provided an explanation regarding the energy results for this complex, as mentioned above. In fact, a linear correlation was obtained between the H⋯N distances vs. De for all the complexes except for the *o*-B_10_H_12_C_2_:NCH one (Figure 4)

As shown in Figure 3, a unique intermolecular bond critical point (BCP) was found in all the complexes, with the exception of the *o*-B_10_H_12_C_2_:NCH complex, where two BCPs were found. The electron density properties at the BCPs are shown in Table S3. In all cases (HB and XB complexes), the values of ρ_BCP_ were small (between 0.016 and 0.010 au), with positive values of Laplacian (▽^2^ρ_BCP_) and total energy (H_BCP_) as an indication of weak interactions in the closed shell regime. An excellent exponential relationship was obtained between the H⋯N distance and ρ_BCP_ in the HB complexes (Figure S1), in agreement with previous reports showing similar relationships [84,85,86].

**Figure 5:**
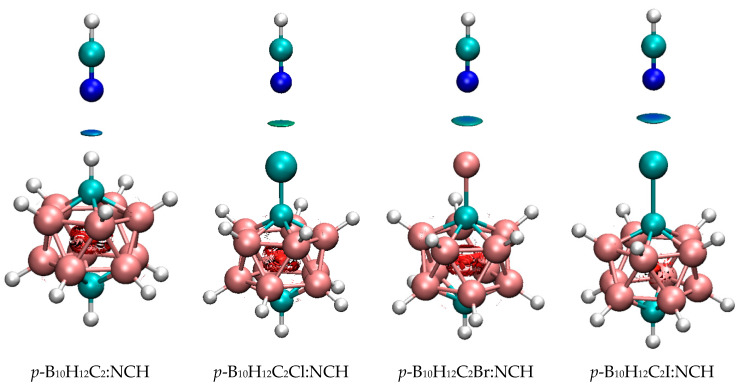
The NCI isosurface (0.05 au) of four of the complexes. The color code indicates strongly attractive (blue), strongly repulsive (red) and weakly attractive or repulsive (green).

The NCI analysis showed the regions with attractive interaction in the intermolecular region (Figure 5). The color of such regions indicates the strength of the interaction. Thus, as the size of the halogen atom increases, the intermolecular region has more blue color, indicating that the interaction was stronger.

**Figure 6:**
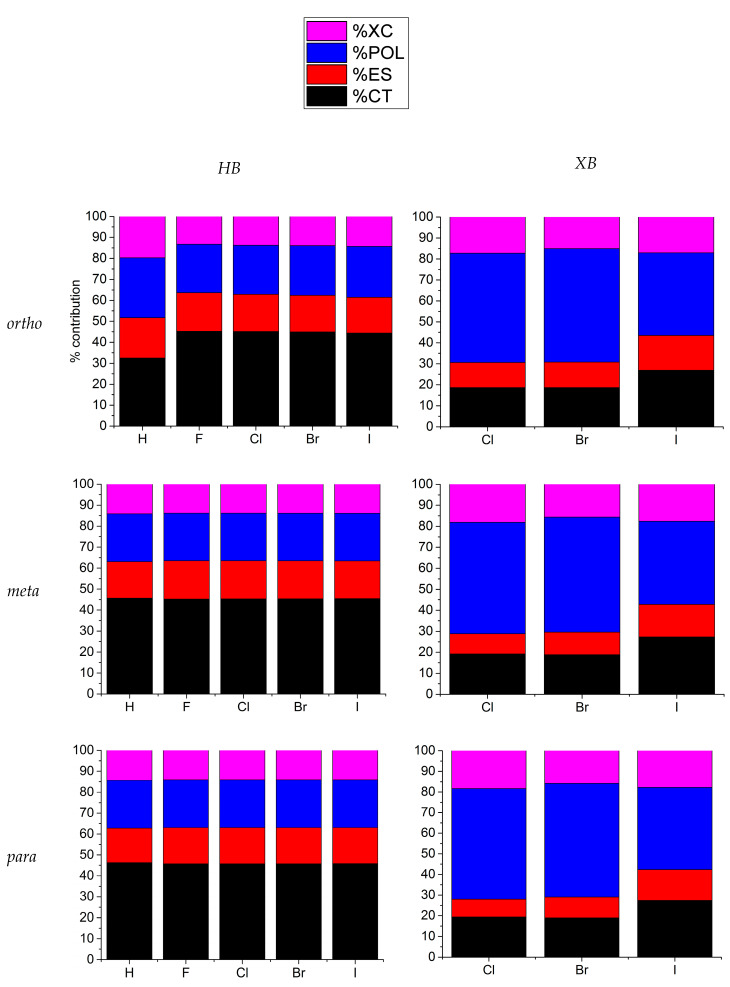
Percentage contribution of the four attractive terms in the NEDA analysis. Magenta, blue, red and black colors correspond to the exchange-correlation, polarization, electrostatic and charge-transfer terms, respectively.

The NEDA partition has been applied to gain insight into the contribution of the different terms to the binding energy. The most important term in the HB complexes was the charge-transfer that corresponds to ~45% of the sum of all the attractive terms (Figure 6 and Table S4). The second most important term was the polarization (~23%), while the electrostatic and exchange terms had smaller contributions (~18% and ~14%, respectively). In contrast, in the XB complexes, the polarization term was the most important, with a ~54% contribution in the chlorine and bromine complexes and a ~40% contribution in the iodine ones, followed by the charge-transfer (19% for chlorine and bromine and 27% for the iodine complexes). In these complexes, the least important contribution corresponded to the electrostatic term (between 9% and 17% of the total attractive terms). Concerning the deformation energies in the HB complexes, the deformation of the NCH was about four times greater as compared to the *closo*-carborane involved in the complex. In the XB complexes, the deformation of the NCH was slightly greater than that of the *closo*-carborane in the chlorine and bromine derivatives, but of a similar magnitude in the iodine derivatives.

**Figure 7:**
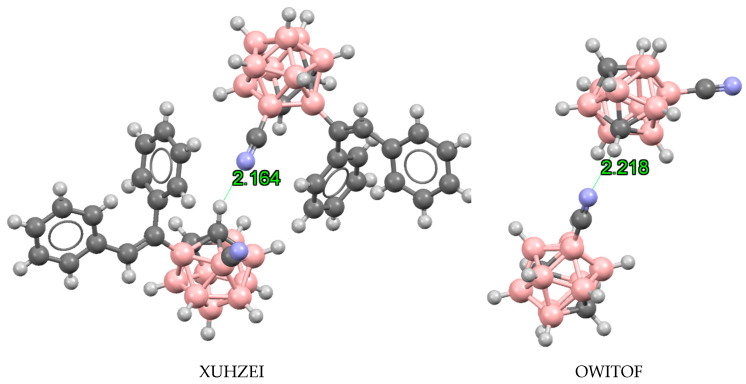
Structures retrieved from the CSD database with an indication of the N⋯H intermolecular distances. The Refcode of the crystal structure is indicated.

The CSD search of *o*-, *m*- and *p*-*closo*-carboranes with C–H⋯N interactions with distances between 1.5 and 3.0 Å provided 70 crystal structures with a total of 113 contacts (Table S5). Some of the shortest ones corresponded to the interaction with a cyano group, such as the two shown in Figure 6. The intermolecular distances found in the two structures depicted in Figure 7 were similar to those found in our calculations for the complexes of 1-halo-*closo*-carboranes with NCH (Table 2).

**Figure 8:**
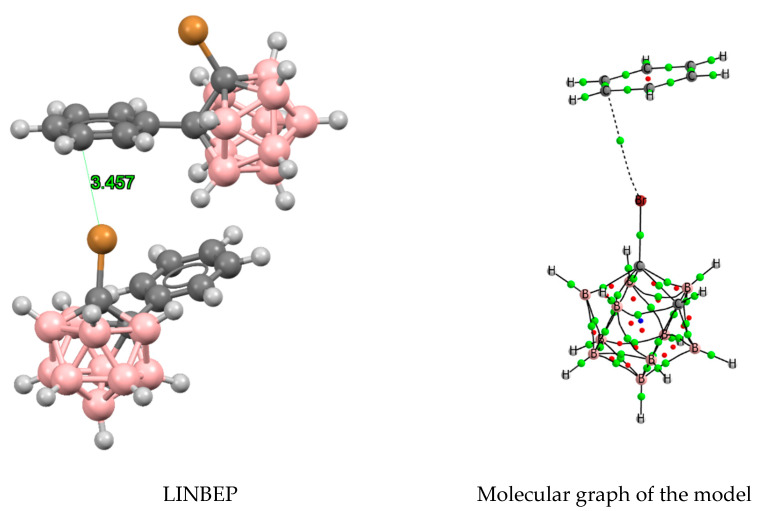
Crystal structure with refcode LINBEP and model calculated at MP2/aug-cc-pVDZ level. The shortest intermolecular Br-C distance in the LIMBEP structure is indicated in Å.

The halo-*closo*-boranes are less frequent in the CSD database, and only three cases have been found where the halogen is a bromine atom. In two of them (Refcodes BRDCBO and KEMYAD), the bromine atoms were interacting with the hydride H-B groups of the carborane of another molecule; in the third structure (LINBEP), the bromine was interacting with the π-cloud of an aromatic ring (Figure 8). A model of this last system has been considered using the same geometry found in the crystal structure. The corresponding dissociation energy is 27 kJ·mol^−1^, and the molecular graph (Figure 8, right) shows a bond critical point linking the bromine atom with the aromatic ring.

### 3.3. Hydrogen and Halogen Bonds in 1-Halobenzene

In this section, the results of the HB and XB complexes between 1-halobenzene and NCH are reported. Only the HBs in *para* disposition have been considered. The energy and geometry results of these complexes are gathered in Table 4. The strength of the hydrogen bonds ranged between 8 and 10 kJ·mol^−1^, while those of the halogen bonds ranged between 4 and 11 kJ·mol^−1^. In line with previous results, the XB and HB complexes of iodobenzene showed similar energy results, while the HB complex was predominant for the rest of the complexes.

The comparison of the HB results of the halobenzenes with those of the *closo*-carboranes indicated that the former were much weaker than the latter (8–10 vs. 17–25 kJ·mol^−1^, respectively). The same was true for the halogen-bonded complexes of the carboranes that were at least between 4 and 6 kJ·mol^−1^ more stable than the corresponding halobenzenes, depending on the halogen atom being considered.

**Figure 9:**
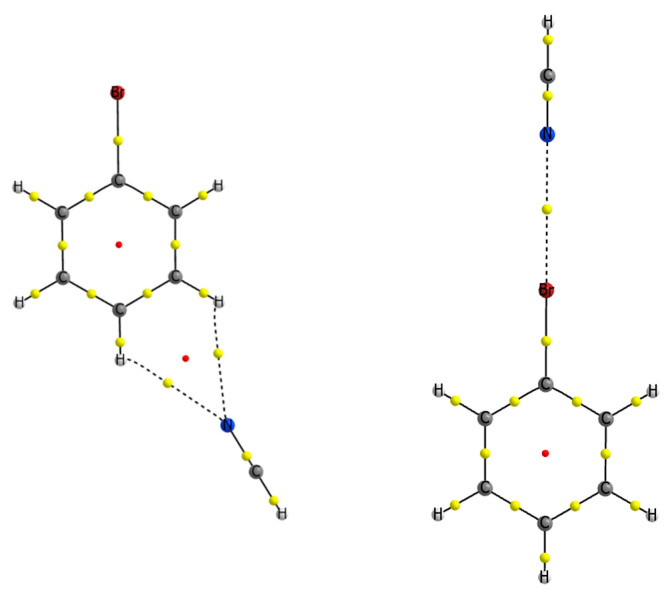
Molecular graph of some of the hydrogen- and halogen-bonded complexes between 1-bromobenzene and NCH. The locations of the bond and ring critical points are indicated with green and red dots, respectively.

Regarding the intermolecular distances, the ones in the C_6_H_5_X:NCH complexes were larger than those of the *closo*-carboranes, especially in the HB complexes, since the former presented bifurcated HB similar to those of the o-B_10_H_12_C_2_:NCH complex (Figure 9).

## 4. Conclusions

In this article, a theoretical study of the hydrogen and halogen bond complexes between 1-halo-*closo*-carboranes and hydrogen cyanide (NCH) has been carried out. The systems have been optimized at the MP2/aug-cc-pVDZ//aug-cc-pVDZ-pp computational level. The complete basis set extrapolation has been used to obtain highly accurate dissociation energies.

The main conclusions of the study are listed below.

The hydrogen bond complexes of the 1-halo-*closo*-carboranes with hydrogen cyanide are more stable than those with halogen bonds, except for in the case of isoenergetic iodine derivatives.

The *ortho* complexes are more stable than the corresponding *meta* ones, with the *para* complexes being the least stable.

In all of the complexes (HB and XB), a single intermolecular bond critical point is found, except for the *o*-B_10_H_12_C_2_:NCH that shows a bifurcated hydrogen bond.

The NEDA analysis shows that while the charge-transfer is the dominant stabilization term in the HB complexes, polarization is the dominant term in the XB complexes.

A CSD search shows 70 crystal structure *closo*-carboranes involving hydrogen bonds with nitrogen bases. Several cases correspond to cyano groups with similar intermolecular distances to the ones obtained in the calculations.

The calculated 1-halobenzene:NCH complexes show smaller dissociation energies than the analogous 1-halocarboranes.

## Figures and Tables

**Table 1:** The MESP V_s,max_ values (au) associated to the atoms bonded to the C atoms (H and X).

	*o*-B_10_H_11_C_2_X		*m*-B_10_H_11_C_2_X		*p*-B_10_H_11_C_2_X	
	H	X	H	X	H	X
X = H	0.068	0.068	0.052	0.052	0.048	0.048
F	0.069	– *	0.059	−0.018	0.054	−0.022
Cl	0.067	0.034	0.058	0.021	0.054	0.017
Br	0.067	0.043	0.058	0.031	0.054	0.027
I	0.065	0.059	0.057	0.047	0.053	0.043

* In this case no V_s,max_ associated to this atom was found.

**Table 2:** Dissociation energy (kJ·mol^−1^) for the hydrogen- (HB) and halogen-bonded (XB) complexes with NCH.

	*o*-B_10_H_11_C_2_X		*m*-B_10_H_11_C_2_X		*p*-B_10_H_11_C_2_X	
	HB	XB	HB	XB	HB	XB
X = H	24.8		17.3		16.0	
F	22.0		18.8		17.3	
Cl	22.1	11.9	18.8	9.2	17.3	8.0
Br	22.0	15.9	18.6	12.8	17.3	11.8
I	22.0	21.8	18.4	18.4	17.0	17.1

**Table 3:** Intermolecular distances (Å) in the HB and XB complexes.

	*o*-B_10_H_11_C_2_X		*m*-B_10_H_11_C_2_X		*p*-B_10_H_11_C_2_X	
	H⋯N (HB)	X⋯N (XB)	H⋯N (HB)	X⋯N (XB)	H⋯N (HB)	X⋯N (XB)
X = H	2.491		2.239		2.255	
F	2.168		2.221		2.242	
Cl	2.162	3.006	2.220	2.982	2.241	3.020
Br	2.159	3.037	2.220	3.012	2.240	3.059
I	2.159	3.050	2.222	3.022	2.240	3.071

**Table 4:** Dissociation energy (kJ·mol^−1^) and intermolecular distances (Å) of the C_6_H_5_X:NCH complexes.

	HB		XB	
	De	N⋯H	De	N⋯X
X = H	8.1	2.720		
F	9.5	2.694		
Cl	10.0	2.689	3.9	3.183
Br	10.2	2.675	6.4	3.148
I	10.3	2.682	10.7	3.223

## Supplementary Materials

The following are available online at https://www.mdpi.com/1996-1944/13/9/2163/s1: Table S1. Electronic energy and optimized geometry of the isolated monomers at MP2/aug-cc-pVDZ/aug-cc-pVDZ-PP computational level, Table S2. Electronic energy and optimized geometry of the 1-halo-*closo-*carboranes:NCH complexes at MP2/aug-cc-pVDZ/aug-cc-pVDZ-PP computational level, Table S3. ρ_BCP_, ▽2ρ_BCP_ and H_BCP_ (au) of the intermolecular BCPs in the 1-halo*-closo-*carboranes:NCH complexes, Figure S1 ρ_BCP_ (au) vs. the interatomic N⋯H distance (Å) in the 1-halo*-closo-*carboranes:NCH (HB) complexes, Table S4. NEDA partition terms, kJ mol^−1^ of the 1-halo-*closo-*carboranes:NCH complexes, Table S5. CH⋯N distances (Å) in the CSD search between *closo*-carboranes and N-bases.
