# Supplementary material for: Hydrogen vs. Halogen Bonds in 1-Halo-Closo-Carboranes
Source: Materials (Basel). 2020 May 7;13(9):2163. doi: 10.3390/ma13092163 (PMC7254210; doi:10.3390/ma13092163)
Supplement: Supplementary file 1 [file materials-13-02163-s001.pdf]

Supplementary Materials

Hydrogen vs. Halogen bonds in 1-halo-closoboranes

Ibon Alkorta[1,*], Jose Elguero[1], Josep M. Oliva-Enrich[2]

Index

Pg. S2–S8 Table S1. Electronic energy and optimized geometry of the isolated monomers at MP2/aug-cc-pVDZ/aug-cc-pVDZ-PP computational level.

Pg. S9–S22 Table S2. Electronic energy and optimized geometry of the 1-halo-*closo*-carboranes:NCH complexes at MP2/aug-cc-pVDZ/aug-cc-pVDZ-PP computational level.

Pg. S23 Table S3. ρ_{BCP}, $\nabla 2\rho_{BCP}$ and H_{BCP} (au) of the intermolecular BCPs in the 1-halo-*closo*-carboranes:NCH complexes.

Pg. S24 Figure S1. ρ_{BCP} (au) vs. the interatomic N···H distance (Å) in the 1-halo-*closo*-carboranes:NCH (HB) complexes.

Pg. S25 Table S4. NEDA partition terms, kJ mol^{-1} of the 1-halo-*closo*-carboranes:NCH complexes.

Pg. S26–S27 Table S5. CH···N distances (Å) in the CSD search between carboranes and N-bases.

Table S1. Electronic energy and optimized geometry of the isolated monomers at MP2/aug-cc-pVDZ/aug-cc-pVDZ-PP computational level.

System	MP2 Energy (Hartree)	Cartesian Coordinates (Å)
o-$B_{10}H_{11}C_2$-1F	−429.97280638	C,0.,0.7437835148,−1.3448219925<br>C,0.,−0.8913918055,−1.2975714022<br>B,1.4750655713,−0.0609198943,−0.9031926022<br>B,0.9023475964,−1.4783116271,0.0295244619<br>B,0.,−0.8719644622,1.4564406388<br>B,−1.466031994,0.0064320412,0.8721568357<br>B,0.,0.9283311444,1.3892937985<br>B,−0.9073084557,1.4276030379,−0.0767096154<br>B,0.9073084557,1.4276030379,−0.0767096154<br>B,−0.9023475964,−1.4783116271,0.0295244619<br>H,−1.4907074702,−2.5048024247,−0.1107071617<br>H,1.4817000645,2.4442606183,−0.3104516857<br>H,0.,−1.4817235357,2.4827742694<br>H,1.4907074702,−2.5048024247,−0.1107071617<br>H,−1.4817000645,2.4442606183,−0.3104516857<br>H,0.,1.6199599405,2.3628451774<br>H,2.3275071373,−0.0702282428,−1.7323145326<br>H,−2.5003763979,0.0298427222,1.467516212<br>H,0.,−1.3762303573,−2.2764449638<br>B,1.466031994,0.0064320412,0.8721568357<br>B,−1.4750655713,−0.0609198943,−0.9031926022<br>H,2.5003763979,0.0298427222,1.467516212<br>H,−2.3275071373,−0.0702282428,−1.7323145326<br>F,0.,1.2887294666,−2.5925029374
o-$B_{10}H_{11}C_2$-1Cl	−789.98805749	C,0.,0.740953494,−1.3432106992<br>C,0.,−0.9011357391,−1.2837970204<br>B,1.4735261724,−0.06771899,−0.8915429021<br>B,0.9012613534,−1.4847191909,0.04464192<br>B,0.,−0.8803370807,1.4731665351<br>B,−1.4650216558,−0.0007238683,0.885886224<br>B,0.,0.9197482855,1.4057196932<br>B,−0.9058363383,1.4204278544,−0.0613292762<br>B,0.9058363383,1.4204278544,−0.0613292762<br>B,−0.9012613534,−1.4847191909,0.04464192<br>H,−1.4929350464,−2.5094247488,−0.0973200832<br>H,1.4845115823,2.4358584017,−0.2904216892<br>H,0.,−1.4914292115,2.4988770033<br>H,1.4929350464,−2.5094247488,−0.0973200832<br>H,−1.4845115823,2.4358584017,−0.2904216892<br>H,0.,1.6121411359,2.3791093811<br>H,2.3379406372,−0.0847394559,−1.707799601<br>H,−2.5013729461,0.0235687388,1.4781044924<br>H,0.,−1.3922985972,−2.2592250307<br>B,1.4650216558,−0.0007238683,0.885886224<br>B,−1.4735261724,−0.06771899,−0.8915429021<br>H,2.5013729461,0.0235687388,1.4781044924

		H,−2.3379406372,−0.0847394559,−1.707799601<br>Cl,0.,1.4745465887,−2.939421618
o-$B_{10}H_{11}C_2$-1Br	−2902.86986278	C,0.,0.7581705491,−1.3326076784<br>C,0.,−0.8840491287,−1.2915461818<br>B,1.4731549799,−0.0544826354,−0.8913205906<br>B,0.9011554276,−1.4822634482,0.0304385665<br>B,0.,−0.8941856755,1.4659016939<br>B,−1.4649304125,−0.0079475873,0.8879653826<br>B,0.,0.9063162406,1.4189430259<br>B,−0.9055171408,1.4237624095,−0.0429583644<br>B,0.9055171408,1.4237624095,−0.0429583644<br>B,−0.9011554276,−1.4822634482,0.0304385665<br>H,−1.4932244406,−2.5050981209,−0.123672175<br>H,1.4858413893,2.4415902717,−0.2572460359<br>H,0.,−1.5170399262,2.4846940104<br>H,1.4932244406,−2.5050981209,−0.123672175<br>H,−1.4858413893,2.4415902717,−0.2572460359<br>H,0.,1.5884695599,2.3996874518<br>H,2.342012414,−0.066197501,−1.7030253707<br>H,−2.5020227765,0.0103453286,1.4792367192<br>H,0.,−1.3679515763,−2.2707250054<br>B,1.4649304125,−0.0079475873,0.8879653826<br>B,−1.4731549799,−0.0544826354,−0.8913205906<br>H,2.5020227765,0.0103453286,1.4792367192<br>H,−2.342012414,−0.066197501,−1.7030253707<br>Br,0.,1.58165828,−3.0555662318
o-$B_{10}H_{11}C_2$-1I	−625.12798071	C,0.,0.784976194,−1.3212264679<br>C,0.,−0.8597346966,−1.3023996169<br>B,1.471731012,−0.0356377862,−0.8909451222<br>B,0.9010091498,−1.4787677826,0.0101359641<br>B,0.,−0.9141256924,1.4552273117<br>B,−1.4647892202,−0.0185398966,0.8905986007<br>B,0.,0.886632695,1.4373682622<br>B,−0.9047382841,1.4276990644,−0.0169227321<br>B,0.9047382841,1.4276990644,−0.0169227321<br>B,−0.9010091498,−1.4787677826,0.0101359641<br>H,−1.4936397615,−2.4987536309,−0.160780715<br>H,1.4883051069,2.4484172448,−0.2094757041<br>H,0.,−1.5535878377,2.4639818528<br>H,1.4936397615,−2.4987536309,−0.160780715<br>H,−1.4883051069,2.4484172448,−0.2094757041<br>H,0.,1.5535666444,2.4286952856<br>H,2.3465922167,−0.0394594201,−1.6966716751<br>H,−2.5026754337,−0.0089730265,1.4809466278<br>H,0.,−1.3343852654,−2.2861432175<br>B,1.4647892202,−0.0185398966,0.8905986007<br>B,−1.471731012,−0.0356377862,−0.8909451222<br>H,2.5026754337,−0.0089730265,1.4809466278<br>H,−2.3465922167,−0.0394594201,−1.6966716751

		I,0.,1.7415532193,−3.2158974402
m-$B_{10}H_{12}C_2$	−330.95472405	C,0.,−1.3089796983,−0.8043405428<br>B,0.9001214611,0.,−1.4225026712<br>B,−0.9001214611,0.,−1.4225026712<br>C,0.,1.3089796983,−0.8043405428<br>B,0.,1.4432576505,0.913493591<br>B,0.9047857162,0.,1.4742776087<br>B,−0.9047857162,0.,1.4742776087<br>B,0.,−1.4432576505,0.913493591<br>B,−1.4591403359,−0.892799674,0.018377352<br>B,1.4591403359,0.892799674,0.018377352<br>H,2.4142767602,1.5978903512,−0.0854170158<br>H,−2.4142767602,−1.5978903512,−0.0854170158<br>H,0.,2.5284761086,1.4080335078<br>H,0.,2.229318079,−1.3928234482<br>H,0.,−2.5284761086,1.4080335078<br>H,−1.5450778069,0.,2.4821815724<br>H,−1.4337911734,0.,−2.4851121272<br>H,1.5450778069,0.,2.4821815724<br>H,1.4337911734,0.,−2.4851121272<br>B,−1.4591403359,0.892799674,0.018377352<br>B,1.4591403359,−0.892799674,0.018377352<br>H,−2.4142767602,1.5978903512,−0.0854170158<br>H,2.4142767602,−1.5978903512,−0.0854170158<br>H,0.,−2.229318079,−1.3928234482
m-$B_{10}H_{11}C_2$-1F	−429.99512783	C,1.3783749504,−1.2556751409,0.0000000021<br>B,1.2079924563,0.1755975877,0.9032589297<br>B,1.2079924562,0.1755975845,−0.9032589304<br>C,−0.0211819751,0.9474012657,−0.0000000017<br>B,−1.5447466111,0.1552016941,−0.0000000002<br>B,−1.2465243588,−1.3639453072,0.9054105703<br>B,−1.2465243589,−1.3639453104,−0.9054105653<br>B,−0.0025866796,−2.2853129248,0.000000004<br>B,0.4595071158,−1.3456911967,−1.4605301297<br>B,−0.4914257754,0.162789393,1.4635942466<br>H,−0.7725593101,0.8420856121,2.4000374891<br>H,0.9252519171,−1.8859924979,−2.4143027769<br>H,−2.5250175409,0.8321305427,−0.0000000013<br>H,0.1595921572,−3.465649749,0.0000000061<br>H,−2.1012978898,−1.8985201353,−1.5440683779<br>H,2.0938218942,0.7676268666,−1.4283226152<br>H,−2.1012978896,−1.8985201298,1.5440683848<br>H,2.0938218944,0.7676268716,1.4283226122<br>B,−0.4914257755,0.1627893878,−1.4635942471<br>B,0.459507116,−1.3456911915,1.4605301344<br>H,−0.7725593103,0.8420856036,−2.400037492<br>H,0.9252519174,−1.8859924894,2.4143027835<br>H,2.3669157615,−1.7204112109,0.0000000029<br>F,−0.0061140675,2.3168059325,−0.0000000041

m-$B_{10}H_{11}C_2$-1Cl	−790.01122761	C,1.3774058236,−1.2730562059,0.000000003<br>B,1.2072250329,0.1607310836,0.9028857707<br>B,1.2072250339,0.1607310804,−0.9028857699<br>C,−0.0184094975,0.941090031,−0.0000000017<br>B,−1.5454343723,0.1384299816,−0.0000000011<br>B,−1.2462863135,−1.3814160685,0.9050025811<br>B,−1.2462863125,−1.3814160717,−0.9050025777<br>B,−0.0024913189,−2.3042183882,0.0000000041<br>B,0.4598955279,−1.3621618812,−1.4596709014<br>B,−0.4922458155,0.1470482606,1.4638496349<br>H,−0.7760872261,0.8180949942,2.4053085938<br>H,0.9255740725,−1.8989664147,−2.4157072706<br>H,−2.5295121461,0.8098727229,−0.0000000029<br>H,0.1593793506,−3.4847973455,0.0000000063<br>H,−2.1021219409,−1.9129202932,−1.5451747493<br>H,2.0968379733,0.7470114545,−1.4279700097<br>H,−2.1021219426,−1.9129202877,1.5451747536<br>H,2.0968379717,0.7470114595,1.4279700094<br>B,−0.4922458138,0.1470482554,−1.463849636<br>B,0.4598955262,−1.362161876,1.4596709068<br>H,−0.7760872234,0.8180949857,−2.4053085977<br>H,0.9255740698,−1.8989664061,2.4157072784<br>H,2.3673775627,−1.7350934175,0.0000000044<br>Cl,0.0008700754,2.7053214015,−0.0000000048
m-$B_{10}H_{11}C_2$-1Br	−2902.89327742	C,1.3771747105,−1.2788780584,0.0000000022<br>B,1.2068726453,0.1555618259,0.9026763479<br>B,1.2068726452,0.1555618227,−0.9026763486<br>C,−0.0181949208,0.9334050719,−0.0000000017<br>B,−1.5455626827,0.1326327342,−0.0000000002<br>B,−1.2461911362,−1.3872573705,0.904884649<br>B,−1.2461911363,−1.3872573738,−0.904884644<br>B,−0.0025432877,−2.309897235,0.0000000041<br>B,0.4599455856,−1.3678213313,−1.4595664097<br>B,−0.4925499383,0.1415762475,1.4638334826<br>H,−0.7773993821,0.8101803354,2.4069972218<br>H,0.9263041826,−1.9049153878,−2.4152731883<br>H,−2.5316380742,0.8014701186,−0.0000000013<br>H,0.1597180497,−3.4906361083,0.0000000062<br>H,−2.1024391559,−1.9188842417,−1.5446916016<br>H,2.0980879999,0.7395328882,−1.4281060359<br>H,−2.1024391557,−1.9188842363,1.5446916086<br>H,2.098088,0.7395328932,1.428106033<br>B,−0.4925499384,0.1415762423,−1.4638334831<br>B,0.4599455857,−1.3678213261,1.4595664145<br>H,−0.7773993824,0.8101803268,−2.4069972246<br>H,0.9263041829,−1.9049153793,2.415273195<br>H,2.3670589012,−1.741481419,0.000000003<br>Br,0.003493805,2.8498300095,−0.0000000051

m-$B_{10}H_{11}C_2$-1I	−625.15158119	C,1.3770969499,−1.2875448261,0.0000000016<br>B,1.2063661818,0.14831969,0.9021986099<br>B,1.2063661809,0.1483196868,−0.9021986117<br>C,−0.0176058831,0.928257338,−0.0000000017<br>B,−1.5451131302,0.1246926907,0.0000000005<br>B,−1.2461951891,−1.3957301308,0.9048970685<br>B,−1.24619519,−1.395730134,−0.9048970623<br>B,−0.0026158001,−2.3187866019,0.0000000041<br>B,0.4602119043,−1.3759370835,−1.4594084319<br>B,−0.4925084713,0.1339775846,1.463466572<br>H,−0.7790785812,0.7966299511,2.4107405396<br>H,0.9266703022,−1.9122555688,−2.4156122279<br>H,−2.5350298124,0.788572335,−0.0000000001<br>H,0.1595535514,−3.4997225434,0.0000000061<br>H,−2.1029500466,−1.9265140517,−1.5449440416<br>H,2.100493188,0.7269108861,−1.4294019772<br>H,−2.102950045,−1.9265140462,1.5449440506<br>H,2.1004931895,0.7269108912,1.4294019725<br>B,−0.4925084728,0.1339775794,−1.463466572<br>B,0.4602119058,−1.3759370782,1.4594084363<br>H,−0.7790785836,0.7966299425,−2.4107405417<br>H,0.9266703046,−1.9122555602,2.4156122337<br>H,2.3674248428,−1.7494298837,0.0000000019<br>I,0.0050388043,3.0555499888,−0.0000000055
p-$B_{10}H_{12}C_2$	−330.96010003	C,−0.0000010904,0.0000007482,−1.5345747802<br>B,0.9009344047,1.240035756,−0.7548242928<br>B,−0.9009426412,1.2400358267,−0.7548261613<br>B,0.0000018809,1.5327653535,0.754822621<br>C,0.0000010903,−0.0000007481,1.5345747802<br>B,0.9009426411,−1.2400358266,0.7548261613<br>B,−0.9009344047,−1.2400357559,0.7548242928<br>B,−0.000001881,−1.5327653534,−0.754822621<br>B,−1.4577498289,−0.4736503151,−0.7548247095<br>B,1.4577498289,0.4736503152,0.7548247095<br>H,2.4145393239,0.7845329417,1.3924400058<br>H,−2.414539324,−0.7845329416,−1.3924400058<br>H,−0.0000006979,0.0000022862,2.6271812622<br>H,0.0000053691,2.5388050818,1.3924384864<br>H,−0.0000053691,−2.5388050817,−1.3924384864<br>H,−1.4922668926,−2.0539373542,1.3924338579<br>H,−1.4922823742,2.0539505892,−1.3924501587<br>H,1.4922823741,−2.0539505892,1.3924501587<br>H,1.4922668925,2.0539373542,−1.3924338579<br>B,−1.4577434416,0.4736507068,0.7548218251<br>B,1.4577434415,−0.4736507068,−0.7548218251<br>H,−2.4145413244,0.7845344681,1.392436565<br>H,2.4145413243,−0.784534468,−1.392436565<br>H,0.0000006978,−0.0000022861,−2.6271812622
p-$B_{10}H_{11}C_2$-1F	−430.00208655	C,0.0000101879,−0.0002571098,−0.9976165119

		B,−0.902919497,1.2427012313,−0.2236518133
		B,−1.4609409098,−0.4747318049,−0.2231760566
		B,−1.457502179,0.4739406045,1.2836192141
		C,−0.0000290598,0.0006031588,2.0639749647
		B,1.4574677933,0.4739499685,1.2836530407
		B,0.9007598412,−1.2394267119,1.2841279011
		B,1.4609434385,−0.4747213139,−0.2231382342
		B,0.0000048962,−1.5361677585,−0.2228608094
		B,−0.0000283171,1.5328743579,1.2833374307
		H,−0.0000408873,2.5403322418,1.9177647754
		H,0.0000172068,−2.5257639453,−0.8837556433
		H,−0.0000495917,0.0009130687,3.1561141081
		H,−2.4154969467,0.7853917479,1.9182351404
		H,2.401942263,−0.7806527908,−0.8842075514
		H,1.4928080998,−2.054151357,1.9190584053
		H,−2.4019243386,−0.7806565776,−0.8842713151
		H,2.4154402662,0.7853902536,1.9182821804
		H,−1.4844808994,2.0429571168,−0.8850454538
		B,−0.9007926257,−1.2394263766,1.2841072299
		B,0.9029092934,1.2427211286,−0.2236339243
		H,−1.4928714166,−2.0541552018,1.9190340211
		H,1.4844716105,2.0429872193,−0.8850185771
		F,0.0000283159,−0.0006433133,−2.3684338126
p-$B_{10}H_{11}C_2$-1Cl	−790.01761467	C,0.0000059954,−0.0002583929,−0.9900016244
		B,−0.9034252633,1.2434322063,−0.2075209494
		B,−1.4617930506,−0.4749962052,−0.207055202
		B,−1.4569040643,0.4737532061,1.3003542842
		C,−0.0000372169,0.0006109678,2.0818333657
		B,1.4568619013,0.4737660251,1.300395973
		B,0.9003813408,−1.2389043066,1.3008667236
		B,1.4617778271,−0.475004203,−0.2070124397
		B,−0.0000011563,−1.5370517887,−0.206743731
		B,−0.0000189076,1.5322482751,1.3000748858
		H,−0.0000385158,2.5412813183,1.9325081754
		H,0.0000209647,−2.5299319498,−0.8624822595
		H,−0.0000523831,0.0009089224,3.1741295873
		H,−2.4164000432,0.7856930516,1.9329670349
		H,2.4059010091,−0.7819553339,−0.8629454924
		H,1.4933834423,−2.0549121018,1.9338064923
		H,−2.405908008,−0.7819402234,−0.8630169187
		H,2.4163268077,0.7856945125,1.9330310194
		H,−1.4869049849,2.0463736104,−0.8637948344
		B,−0.900423187,−1.2389210785,1.3008389423
		B,0.9034323369,1.2434221797,−0.2074989614
		H,−1.4934248348,−2.0549321462,1.9337763834
		H,1.4869404063,2.046362515,−0.8637499694
		Cl,0.0000299669,−0.0007400223,−2.7563257377
p-$B_{10}H_{11}C_2$-1Br	−2902.89950893	C,0.000034925,−0.0002957799,−0.983180813
		B,−0.9035228541,1.2435605163,−0.2028240022

		B,−1.4619386447,−0.475072109,−0.2022935037
		B,−1.4570160376,0.4738307153,1.305602907
		C,−0.0000691836,0.0006886992,2.0869516272
		B,1.4569321278,0.4738449847,1.3056969914
		B,0.900422269,−1.2389364662,1.3062274617
		B,1.4619494307,−0.4750585183,−0.2021976724
		B,0.0000158986,−1.5372420186,−0.2019035349
		B,−0.0000478056,1.5323936388,1.3053126474
		H,−0.0000813047,2.5416423869,1.9375524531
		H,0.0000513079,−2.5323978172,−0.8545330393
		H,−0.0000957399,0.0010256277,3.1793311572
		H,−2.4167017609,0.7858492188,1.9380146407
		H,2.4082284433,−0.7827116543,−0.8550012353
		H,1.4935038669,−2.0550511378,1.9390824135
		H,−2.4081672768,−0.7827310921,−0.8551600606
		H,2.4165629461,0.7858509807,1.9381988727
		H,−1.4883185943,2.04826205,−0.8560379701
		B,−0.9004962026,−1.2389441063,1.3061693894
		B,0.9035437114,1.2435740776,−0.2027585237
		H,−1.4936184522,−2.0550724332,1.9389665968
		H,1.4883741138,2.0482909351,−0.8559427176
		Br,0.0001005753,−0.0009076124,−2.9019796114
		C,0.000036641,−0.0002855828,−0.9779512172
		B,−0.9031167126,1.2429824877,−0.1954021268
		B,−1.4612570424,−0.4748475923,−0.1948655376
		B,−1.4573008328,0.4739229496,1.3138122861
		C,−0.0000647218,0.0006848272,2.0945737308
		B,1.457218297,0.4739295354,1.3139112152
		B,0.9005920034,−1.239184517,1.3144432673
		B,1.4612681769,−0.4748304892,−0.1947535668
		B,0.0000079689,−1.5365092161,−0.1944598926
		B,−0.0000560718,1.5326927549,1.313526911
		H,−0.000076809,2.5417080038,1.946328127
p-$B_{10}H_{11}C_2$-1I	−625.15763839	H,0.0000407741,−2.5355773294,−0.8418870225
		H,−0.0000928743,0.0010312671,3.1871908383
		H,−2.4167719414,0.7858581917,1.946815661
		H,2.4112761825,−0.7837015512,−0.8423923887
		H,1.4935537606,−2.0551571172,1.9478723873
		H,−2.4112105342,−0.7837257374,−0.842555544
		H,2.4166393527,0.7858832244,1.9469720521
		H,−1.4902325366,2.0508818892,−0.8434443337
		B,−0.9006664229,−1.2391733645,1.3143768228
		B,0.9031167203,1.2430013419,−0.1953224444
		H,−1.493652242,−2.0551135117,1.9477573338
		H,1.4902680981,2.0509025919,−0.8433225133
		I,0.0001265251,−0.0009799702,−3.1079295711

Table S2. Electronic energy and optimized geometry of the 1-halo-*closo*-carboranes:NCH complexes at MP2/aug-cc-pVDZ/aug-cc-pVDZ-PP computational level.

System	MP2 Energy (Hartree)	Cartesian Coordinates (Å)
o-$B_{10}H_{12}C_2$:NCH (HB)	−424.12580502	C,0.,0.3008301696,−1.4893367427<br>C,0.,−1.2771953985,−1.0727357017<br>B,1.4676947288,−0.3823578796,−0.8801684831<br>B,0.9009029751,−1.5588479005,0.3487564555<br>B,0.,−0.6559098517,1.6111423891<br>B,−1.4658216274,0.072682044,0.8436000788<br>B,0.,1.085301937,1.1514958666<br>B,−0.9009385149,1.2474193719,−0.3921030116<br>B,0.9009385149,1.2474193719,−0.3921030116<br>B,−0.9009029751,−1.5588479005,0.3487564555<br>H,−1.4887877695,−2.5919153269,0.4420975166<br>H,1.4888208961,2.1919524102,−0.8208248559<br>H,0.,−1.0267767044,2.7473221441<br>H,1.4887877695,−2.5919153269,0.4420975166<br>H,−1.4888208961,2.1919524102,−0.8208248559<br>H,0.,1.9686311257,1.9565872481<br>H,2.3408229941,−0.5887161224,−1.662134153<br>H,−2.5004223261,0.2251652971,1.4211104701<br>H,0.,−1.968384308,−1.9160371991<br>B,1.4658216274,0.072682044,0.8436000788<br>B,−1.4676947288,−0.3823578796,−0.8801684831<br>H,2.5004223261,0.2251652971,1.4211104701<br>H,−2.3408229941,−0.5887161224,−1.662134153<br>H,0.,0.485736981,−2.5639293735<br>N,0.,−1.2865514371,−4.3130047525<br>C,0.,−1.5852965335,−5.4563311366<br>H,0.,−1.8577164445,−6.4991842626
o-$B_{10}H_{11}C_2$-1F:NCH (HB)	−523.16656691	C,0.,0.744747693,−1.3419371713<br>C,0.,−0.8893194779,−1.3009372604<br>B,1.47263989,−0.0595902891,−0.9033185625<br>B,0.9008519101,−1.4755852401,0.0268237816<br>B,0.,−0.8722044473,1.4560982627<br>B,−1.4658876322,0.006781369,0.8731567519<br>B,0.,0.9278828388,1.391793149<br>B,−0.9070677209,1.4285090219,−0.0734127791<br>B,0.9070677209,1.4285090219,−0.0734127791<br>B,−0.9008519101,−1.4755852401,0.0268237816<br>H,−1.490009912,−2.502020868,−0.1117696262<br>H,1.4803689658,2.4469199229,−0.303803571<br>H,0.,−1.4824649782,2.4824681823<br>H,1.490009912,−2.502020868,−0.1117696262<br>H,−1.4803689658,2.4469199229,−0.303803571<br>H,0.,1.6183442879,2.3667124701<br>H,2.3268003805,−0.0660570073,−1.7310595765<br>H,−2.499487631,0.0298520211,1.4706506514

		H,0.,−1.3720662386,−2.2826496611<br>B,1.4658876322,0.006781369,0.8731567519<br>B,−1.47263989,−0.0595902891,−0.9033185625<br>H,2.499487631,0.0298520211,1.4706506514<br>H,−2.3268003805,−0.0660570073,−1.7310595765<br>F,0.,1.2980441504,−2.5864988285<br>N,0.,−2.2659800405,−4.2573752124<br>C,0.,−2.73957121,−5.3391412045<br>H,0.,−3.1716052886,−6.3263846332
o-$B_{10}H_{11}C_2$-1Cl:NCH (HB)	−883.18195058	C,0.,0.7383586098,−1.3423406042<br>C,0.,−0.9023262277,−1.2863259725<br>B,1.4711671942,−0.0694476224,−0.8927245613<br>B,0.8998619306,−1.4837991602,0.0434005574<br>B,0.,−0.8796023795,1.4733457214<br>B,−1.4645428421,−0.0005810986,0.8857887277<br>B,0.,0.9200617534,1.4058390675<br>B,−0.9053807945,1.4197199235,−0.0610152128<br>B,0.9053807945,1.4197199235,−0.0610152128<br>B,−0.8998619306,−1.4837991602,0.0434005574<br>H,−1.491968635,−2.5086732466,−0.0966347316<br>H,1.4837087292,2.436192892,−0.2884778686<br>H,0.,−1.4893612345,2.5003221638<br>H,1.491968635,−2.5086732466,−0.0966347316<br>H,−1.4837087292,2.436192892,−0.2884778686<br>H,0.,1.6129258508,2.3794499511<br>H,2.3365996396,−0.0859918264,−1.708291885<br>H,−2.5007578399,0.0246494604,1.4792220813<br>H,0.,−1.3931041977,−2.2637044749<br>B,1.4645428421,−0.0005810986,0.8857887277<br>B,−1.4711671942,−0.0694476224,−0.8927245613<br>H,2.5007578399,0.0246494604,1.4792220813<br>H,−2.3365996396,−0.0859918264,−1.708291885<br>Cl,0.,1.4761555402,−2.9374636512<br>N,0.,−2.2592865902,−4.2446336636<br>C,0.,−2.7249450575,−5.3299513581<br>H,0.,−3.1495895597,−6.3203891926
o-$B_{10}H_{11}C_2$-1Cl:NCH (XB)	−883.17706351	C,−0.0005716255,0.7968612737,−1.2848217997<br>C,0.008427416,−0.8468674735,−1.2683317022<br>B,1.4750401946,−0.0183670592,−0.8486810706<br>B,0.909125215,−1.4656237924,0.0442853619<br>B,0.0007730719,−0.9084843961,1.4875877601<br>B,−1.4668392674,−0.0202129984,0.921782976<br>B,−0.009100171,0.892553451,1.4724354889<br>B,−0.9126302855,1.4302912678,0.0173454316<br>B,0.8968814653,1.4402483792,0.0225970289<br>B,−0.8930002061,−1.4755426183,0.0390553691<br>H,−1.4777985328,−2.4996561329,−0.1344811408<br>H,1.4715964694,2.4653727285,−0.1718575527<br>H,0.001385029,−1.5492332256,2.4955016491

		H,1.5061723621,−2.4832306916,−0.125822492
		H,−1.4974468936,2.4490363125,−0.1804905388
		H,−0.0156254216,1.5553354298,2.4665773091
		H,2.3426754966,−0.0087017779,−1.6618225454
		H,−2.5048954617,−0.019130196,1.5123473441
		H,0.0138405734,−1.3092794867,−2.2578366628
		B,1.4617736769,−0.0040872127,0.9302801578
		B,−1.4696279583,−0.0345845931,−0.8572179466
		H,2.4963077052,0.0084138999,1.5268566438
		H,−2.3325802483,−0.0344607492,−1.6753782481
		Cl,−0.0002929093,1.5727654879,−2.8577677008
		N,−0.000462623,2.9129870684,−5.5487369998
		C,−0.0012013108,3.5021816262,−6.5736535388
		H,−0.0019257604,4.0402382048,−7.5069828542
		C,0.,0.755660033,−1.3315337279
		C,0.,−0.8850382831,−1.294055234
		B,1.4709155464,−0.0560986508,−0.8925124299
		B,0.8998113276,−1.481274859,0.0291284574
		B,0.,−0.8934979871,1.4659910034
		B,−1.4644974706,−0.0077998617,0.8878776354
		B,0.,0.9066087449,1.4190612047
		B,−0.9051420671,1.4231872295,−0.042680584
		B,0.9051420671,1.4231872295,−0.042680584
		B,−0.8998113276,−1.481274859,0.0291284574
		H,−1.4922177229,−2.5043686834,−0.1231175635
		H,1.4851255661,2.4420039507,−0.2553798033
		H,0.,−1.5151349675,2.4860139577
o-$B_{10}H_{11}C_2$-1Br:NCH (HB)	−2996.06392077	H,1.4922177229,−2.5043686834,−0.1231175635
		H,−1.4851255661,2.4420039507,−0.2553798033
		H,0.,1.5891593355,2.4000611894
		H,2.3407068588,−0.0673167509,−1.7035992742
		H,−2.5014157158,0.0113875502,1.4804512206
		H,0.,−1.3692323281,−2.2746909414
		B,1.4644974706,−0.0077998617,0.8878776354
		B,−1.4709155464,−0.0560986508,−0.8925124299
		H,2.5014157158,0.0113875502,1.4804512206
		H,−2.3407068588,−0.0673167509,−1.7035992742
		Br,0.,1.5828413569,−3.0535654194
		N,0.,−2.2756252172,−4.2340139382
		C,0.,−2.7737844464,−5.3048464997
		H,0.,−3.227970093,−6.2820749101
		C,−0.0011103478,0.781155444,−1.2716224966
		C,0.0084641829,−0.8622516032,−1.2484708104
		B,1.4738458312,−0.0310297111,−0.8338209547
o-$B_{10}H_{11}C_2$-1Br:NCH (XB)	−2996.06104133	B,0.9096086439,−1.4759311674,0.0662404794
		B,0.0015437061,−0.9143091051,1.508037559
		B,−1.4664892762,−0.028363766,0.9388080305
		B,−0.0089539354,0.8865174039,1.4868454906
		B,−0.9124192725,1.4189221732,0.0292292909

		B,0.8958379232,1.4295251482,0.0339738927
		B,−0.8923066293,−1.4864927332,0.0615112213
		H,−1.4773491606,−2.5111856072,−0.1087342274
		H,1.4720456793,2.4540153655,−0.1605085777
		H,0.0026255775,−1.5518610784,2.518203757
		H,1.5075125685,−2.4936861907,−0.1008982098
		H,−1.499578151,2.436592511,−0.1683026863
		H,−0.0154620112,1.5531771974,2.4786042381
		H,2.346218012,−0.0279955567,−1.6421310245
		H,−2.5050831536,−0.0250651053,1.5285893825
		H,0.0138140505,−1.3323346927,−2.2344822533
		B,1.462054566,−0.0112054502,0.9464920906
		B,−1.4687157017,−0.0482754732,−0.8415454394
		H,2.4974339186,0.0042510504,1.5417109281
		H,−2.3368053859,−0.0554335501,−1.654435778
		Br,−0.0015769498,1.6269759122,−2.9833137161
		N,−0.0021795943,2.9806329285,−5.6406114332
		C,−0.0016884401,3.5680774776,−6.6660793655
		H,−0.0012886504,4.1044031787,−7.6005223881
		C,0.,0.7824180856,−1.3200752045
		C,0.,−0.8607517984,−1.3048410333
		B,1.4695223309,−0.0370924205,−0.8921790639
		B,0.8997474116,−1.4777012264,0.0087708535
		B,0.,−0.9134608,1.4553105313
		B,−1.4644180953,−0.0183214945,0.8904711238
		B,0.,0.8869129431,1.4375284729
		B,−0.9043587242,1.4272222325,−0.0165215644
		B,0.9043587242,1.4272222325,−0.0165215644
		B,−0.8997474116,−1.4777012264,0.0087708535
		H,−1.4926181155,−2.4980210351,−0.1604505586
		H,1.4874405358,2.4489090013,−0.207553462
		H,0.,−1.551785756,2.4652930512
o-$B_{10}H_{11}C_2$-1I:NCH (HB)	−718.32207036	H,1.4926181155,−2.4980210351,−0.1604505586
		H,−1.4874405358,2.4489090013,−0.207553462
		H,0.,1.5541935487,2.4291203309
		H,2.3449028982,−0.0403207066,−1.697719448
		H,−2.5021481281,−0.0078630265,1.4820941352
		H,0.,−1.3369694056,−2.2892615995
		B,1.4644180953,−0.0183214945,0.8904711238
		B,−1.4695223309,−0.0370924205,−0.8921790639
		H,2.5021481281,−0.0078630265,1.4820941352
		H,−2.3449028982,−0.0403207066,−1.697719448
		I,0.,1.7426853267,−3.2135221254
		N,0.,−2.2864780059,−4.2281163793
		C,0.,−2.8444035352,−5.2691494425
		H,0.,−3.3525483404,−6.219423164
		C,−0.0007916369,0.7631048329,−1.2464174788
o-$B_{10}H_{11}C_2$-1I:NCH (XB)	−718.32129216	C,0.0081217572,−0.8822296929,−1.2154249741
		B,1.4718924916,−0.0499947128,−0.8046013696

		B,0.9089453574,−1.4941168605,0.1005353852<br>B,0.0013553512,−0.9301645853,1.5417821894<br>B,−1.4661819207,−0.0443928446,0.9701571312<br>B,−0.0084274531,0.8703357015,1.5179924385<br>B,−0.9105730996,1.4006891423,0.0582773138<br>B,0.8952824302,1.4105590507,0.0628913617<br>B,−0.8926430167,−1.5039839653,0.0959370032<br>H,−1.4785248193,−2.5286581097,−0.0727106904<br>H,1.4754272349,2.4341428538,−0.1271098377<br>H,0.0022593935,−1.5666049517,2.5530475963<br>H,1.506862826,−2.5123057437,−0.0650937987<br>H,−1.5008875568,2.4178821965,−0.1347179816<br>H,−0.0145924518,1.5388753027,2.5087154215<br>H,2.3511475218,−0.0543290638,−1.6059821538<br>H,−2.5055725062,−0.0390010421,1.5588659563<br>H,0.0132401188,−1.3608431815,−2.1973232091<br>B,1.4620102091,−0.0283798735,0.9776209725<br>B,−1.4667444942,−0.0660976205,−0.8120833166<br>H,2.4982673582,−0.0116386766,1.571615783<br>H,−2.3418107866,−0.0800253237,−1.6179426508<br>I,−0.0011166172,1.7116985916,−3.1492492525<br>N,−0.003384431,3.0946619871,−5.8336783801<br>C,−0.0022041008,3.6817479942,−6.8587021345<br>H,−0.0013591592,4.2178935952,−7.7936343246
m-$B_{10}H_{12}C_2$:NCH (HB)	−424.14644076	C,1.4237189644,−1.2056645654,−0.0065439747<br>B,1.2139167312,0.2225300937,0.8969881133<br>B,1.2103906182,0.2280282842,−0.9004884437<br>C,−0.0293815246,0.9728671522,0.002985951<br>B,−1.5335055369,0.1338456744,0.0033782237<br>B,−1.1983707297,−1.3804827164,0.9027354955<br>B,−1.2019215226,−1.3749562934,−0.9065146626<br>B,0.067708018,−2.2673781893,−0.0071152211<br>B,0.5045328734,−1.3099170978,−1.4626127498<br>B,−0.4799985357,0.1669174621,1.460502166<br>H,−0.7836930918,0.8107207861,2.4165466368<br>H,0.9785811962,−1.8370412023,−2.4205862187<br>H,−2.5458287795,0.7647063666,0.0072943498<br>H,0.2566856752,−3.4449640191,−0.0110662352<br>H,−2.0437222847,−1.9308408389,−1.5460300876<br>H,2.0914062581,0.8210295571,−1.4352635708<br>H,−2.0376492365,−1.9402801673,1.5421575154<br>H,2.097058996,0.8122310315,1.4319214804<br>B,−0.4857328423,0.1758392257,−1.4576381292<br>B,0.5102601447,−1.3188140349,1.4524670285<br>H,−0.7932152773,0.8254548579,−2.4085235156<br>H,0.9880759196,−1.8518046879,2.4053240688<br>H,2.4230288175,−1.6474978817,−0.009892383<br>H,−0.0514471256,2.0649573559,0.0063634091<br>N,4.4315364295,−2.6360252095,−0.0121224805<br>C,5.411450677,−3.2958747567,−0.003394281

		H,6.305468168,−3.8974291867,0.0044145153
m-$B_{10}H_{11}C_2$-1F:NCH (HB)	−523.18760279	C,1.407673552,−1.2547220497,−0.0032301608<br>B,1.226214898,0.1727826606,0.9018046138<br>B,1.2253901535,0.1769748335,−0.9013952861<br>C,−0.0095597651,0.9397761882,0.0025362238<br>B,−1.5277088101,0.13780508,0.0013584767<br>B,−1.218640145,−1.38104277,0.9029319955<br>B,−1.2194792338,−1.3768243966,−0.9075515338<br>B,0.0327392714,−2.2904184035,−0.0050033496<br>B,0.4872919045,−1.3457529972,−1.4617211783<br>B,−0.4734890241,0.1491034216,1.4641600905<br>H,−0.7599318654,0.8251097087,2.4017581678<br>H,0.9533527171,−1.8806559091,−2.4186294532<br>H,−2.512833721,0.8082437147,0.0033649525<br>H,0.1997784961,−3.4702157774,−0.0078355741<br>H,−2.0720523854,−1.9152924115,−1.5466786543<br>H,2.1046733832,0.7791972555,−1.426171182<br>H,−2.070628846,−1.922488017,1.5403231561<br>H,2.1059802775,0.7725471564,1.4285992774<br>B,−0.4748286621,0.1559145108,−1.4623270015<br>B,0.4886444296,−1.3525370038,1.4556762425<br>H,−0.7621150048,0.836270888,−2.3965165782<br>H,0.9555836163,−1.8918847732,2.4096560953<br>H,2.4002690387,−1.712073697,−0.0047414318<br>F,−0.0054305729,2.3104323447,0.0057238585<br>N,4.4203147482,−2.6348765589,−0.0069896351<br>C,5.4460858617,−3.2206074114,−0.0103810282<br>H,6.3820611128,−3.7546086332,−0.0134345314
m-$B_{10}H_{11}C_2$-1Cl: NCH (HB)	−883.20365011	C,1.405849264,−1.2719694069,−0.0022996454<br>B,1.2255627383,0.1590578655,0.9001975793<br>B,1.2238588818,0.1602489923,−0.9026691012<br>C,−0.0073393361,0.9328946128,0.0004392283<br>B,−1.5289484899,0.121576005,0.0012662012<br>B,−1.2185721966,−1.3962382244,0.9046283019<br>B,−1.2201784172,−1.3950896735,−0.9045811658<br>B,0.0316877299,−2.308922995,−0.0016387228<br>B,0.4861683445,−1.3648132054,−1.4590556414<br>B,−0.4740542291,0.1359586241,1.4639421814<br>H,−0.7626435733,0.8064619866,2.4048086126<br>H,0.9524293065,−1.8986820532,−2.416722668<br>H,−2.5173950223,0.7873231611,0.0026469251<br>H,0.1986203204,−3.4890416558,−0.0026193217<br>H,−2.0744818271,−1.9316569996,−1.5434967574<br>H,2.1066916547,0.7557669749,−1.4291781524<br>H,−2.0717211785,−1.9336437208,1.5443607797<br>H,2.1093644469,0.7539288624,1.4258565968<br>B,−0.4767497505,0.1378678281,−1.4633549516<br>B,0.4888540223,−1.3666400737,1.456077051<br>H,−0.7671083189,0.8096080769,−2.4027823746

		H,0.9568617158,−1.9017892874,2.4121811635
		H,2.3994392545,−1.727674991,−0.0036241283
		Cl,−0.0010608252,2.6984391324,0.0015946812
		N,4.4224334961,−2.6413207488,−0.0072023507
		C,5.4513935896,−3.2213799033,−0.0138075315
		H,6.3903938242,−3.7501122301,−0.0196802173
		C,1.3747234115,−1.3135122724,0.0021585903
		B,1.2121620985,0.1220279852,0.9042144682
		B,1.2151909098,0.1223850787,−0.8998554756
		C,−0.0055484936,0.9137533983,0.0002982169
		B,−1.5367862977,0.1178597874,−0.0024251937
		B,−1.2501170545,−1.404344962,0.9023811263
		B,−1.2470988804,−1.4039801879,−0.9068813003
		B,−0.0115242908,−2.3352649795,−0.0003719178
		B,0.4593596858,−1.3958672011,−1.4585747898
		B,−0.4870786958,0.1191872314,1.4621667949
		H,−0.7687565355,0.7907020733,2.4041301091
		H,0.9236662827,−1.9374686957,−2.4129411751
		H,−2.5176939888,0.7942628778,−0.0039418577
m-$B_{10}H_{11}C_2$-1Cl: NCH (XB)	−883.19916280	H,0.1428443629,−3.5171877193,−0.0003535965
		H,−2.1052949669,−1.9315483253,−1.5478793976
		H,2.1107515559,0.7005510998,−1.4241368985
		H,−2.110452136,−1.9321854809,1.5402827559
		H,2.1059499364,0.6999904484,1.4317346284
		B,−0.4821696305,0.1197856886,−1.4634879302
		B,0.4544744909,−1.3964556429,1.4597739515
		H,−0.7607098107,0.7916745293,−2.4061231501
		H,0.9155814228,−1.9384392992,2.4154721809
		H,2.3610137554,−1.7833394111,0.0037221119
		Cl,0.025055398,2.6759150823,0.0007387583
		N,0.0605740047,5.7127665037,0.00124527
		C,0.02869511,6.8947783072,0.000463815
		H,−0.0011554184,7.971582383,−0.0002634818
		C,1.4053162035,−1.278482521,−0.0043631047
		B,1.2260464856,0.1532250435,0.89862781
		B,1.2228056678,0.1551337403,−0.9037181961
		C,−0.0071673662,0.9256657945,0.0005026501
		B,−1.5292294551,0.1163117151,0.0023919156
		B,−1.218350078,−1.4023821085,0.9049344883
		B,−1.2216022076,−1.400453465,−0.9044896073
m-$B_{10}H_{11}C_2$-1Br: NCH (HB)	−2996.08571144	B,0.0308375982,−2.3150506558,−0.0029976621
		B,0.4845224491,−1.3700937391,−1.4603587265
		B,−0.4730183859,0.130127949,1.4642318393
		H,−0.7614881408,0.7967599916,2.4080580479
		H,0.9497969212,−1.9029396393,−2.4191774453
		H,−2.519737126,0.7791979173,0.0048721838
		H,0.197314745,−3.4953969813,−0.0045580967
		H,−2.0769950997,−1.9358137859,−1.5430998652
		H,2.1069482076,0.7480847875,−1.4312766652

		H,−2.0714426778,−1.9390955173,1.5454705663<br>H,2.1120679571,0.7450573896,1.4242765432<br>B,−0.4782979734,0.1332423482,−1.4632082571<br>B,0.4897626448,−1.373191935,1.454731378<br>H,−0.7701677707,0.8018657421,−2.4045788344<br>H,0.9584791027,−1.9080663413,2.410740528<br>H,2.3990843261,−1.7339289611,−0.0065866957<br>Br,0.0015046423,2.8429071913,0.0025249327<br>N,4.4240932484,−2.6430545891,−0.0062833877<br>C,5.4541534655,−3.2212637949,−0.0093477021<br>H,6.394118041,−3.7482086222,−0.0120320652
m-$B_{10}H_{11}C_2$-1Br: NCH (XB)	−2996.08327217	C,1.3746404336,−1.3333352341,0.0003350692<br>B,1.2126398142,0.1037727973,0.9018907847<br>B,1.213296984,0.1039212612,−0.9010833376<br>C,−0.0055685395,0.8935793344,0.0000311096<br>B,−1.5367715945,0.098488994,−0.0006001346<br>B,−1.2489581698,−1.4241562417,0.9040211185<br>B,−1.2482922977,−1.4240088687,−0.9052598518<br>B,−0.0114586967,−2.3552000889,−0.0002418353<br>B,0.457678392,−1.4154752153,−1.459067378<br>B,−0.4853742055,0.1005514352,1.4621321735<br>H,−0.767083543,0.768256188,2.4071429829<br>H,0.9208691854,−1.9565607539,−2.4144037022<br>H,−2.5203473419,0.7714252772,−0.0009096054<br>H,0.1429575201,−3.5372995761,−0.0002838878<br>H,−2.1074954903,−1.9513831358,−1.5452771915<br>H,2.1100282962,0.6789937468,−1.4273255141<br>H,−2.1086191873,−1.9516388792,1.5433328611<br>H,2.1089880604,0.6787510397,1.4288843272<br>B,−0.4843098008,0.1007917571,−1.4625652437<br>B,0.4566194033,−1.4157074728,1.4590662391<br>H,−0.7653374018,0.768658804,−2.4076639335<br>H,0.9191158536,−1.9569631935,2.4146436244<br>H,2.3612472261,−1.8026718333,0.0006578111<br>Br,0.0286366624,2.8098092612,0.000210967<br>N,0.0598704138,5.8216051763,0.0008303182<br>C,0.0287859025,7.0032505573,0.0013649361<br>H,−0.0001016529,8.080173161,0.0016839058
m-$B_{10}H_{11}C_2$-1I:NCH (HB)	−718.34395097	C,1.4081940349,−1.2800393581,−0.0034918208<br>B,1.2232730479,0.1528403005,0.8987764732<br>B,1.2207133803,0.1543948532,−0.9026842165<br>C,−0.0117217301,0.9226224067,0.0004905227<br>B,−1.5309376294,0.1050510808,0.001956475<br>B,−1.2151044991,−1.412958793,0.9048417148<br>B,−1.2177177711,−1.4113651737,−0.9044885113<br>B,0.0374855568,−2.3216733461,−0.0024336006<br>B,0.4886826213,−1.3745763374,−1.4595253965<br>B,−0.4754938893,0.1233887164,1.4638358854<br>H,−0.7684286184,0.7829945916,2.4116558461

		H,0.9563221127,−1.9049843044,−2.418670509
		H,−2.5275727599,0.7593899752,0.0039283714
		H,0.2081134975,−3.50159939,−0.003694833
		H,−2.0714496839,−1.9489355498,−1.5436804839
		H,2.1057841615,0.7451139565,−1.4319639344
		H,−2.0670100314,−1.951637542,1.5455261578
		H,2.1099108439,0.7426066535,1.4265083907
		B,−0.4796836867,0.1259400554,−1.4629061058
		B,0.4928745407,−1.3771345555,1.4550075238
		H,−0.7752979087,0.7872369768,−2.4087146377
		H,0.9632859567,−1.9091953905,2.4118770203
		H,2.4038517396,−1.7314463548,−0.0053054073
		I,−0.0112943399,3.0505631286,0.0023311403
		N,4.4255624873,−2.6526488356,−0.0088983322
		C,5.4507534392,−3.2394963207,−0.0113766203
		H,6.3862605523,−3.7742944906,−0.0136145404
		C,1.3739218226,−1.372277324,−0.0058675405
		B,1.2167112339,0.0670184796,0.8955379301
		B,1.209429143,0.0673075584,−0.9055006485
		C,−0.0033230088,0.8606501352,0.0000842117
		B,−1.5350499269,0.0634318875,0.0061147834
		B,−1.2455029814,−1.460219027,0.9093234712
		B,−1.2528054361,−1.4599449604,−0.8998732465
		B,−0.0130520245,−2.3927576447,−0.0004105779
		B,0.451027976,−1.45252788,−1.4609038511
		B,−0.4778066151,0.0649752185,1.4632825512
		H,−0.7567215923,0.725913339,2.4143840787
		H,0.9095114793,−1.9932231551,−2.418890306
		H,−2.5224678661,0.7315643795,0.0101892362
m-$B_{10}H_{11}C_2$-1I:NCH (XB)	−718.34362739	H,0.140100601,−3.5752991221,−0.0012140665
		H,−2.1156306202,−1.9857629951,−1.5365335074
		H,2.1076875314,0.6351925701,−1.4378828134
		H,−2.1031771358,−1.9862215712,1.5527696038
		H,2.1192546541,0.6347107447,1.4208125394
		B,−0.4896247359,0.0654195722,−1.4595358908
		B,0.4628090444,−1.4529951478,1.4565728937
		H,−0.7761907251,0.7267028545,−2.4081304324
		H,0.9290274355,−1.9939901287,2.4106529731
		H,2.3604237563,−1.8420413497,−0.0099220987
		I,0.0349187578,2.9917207665,0.0000727369
		N,0.0580304391,6.0502709519,0.0019985886
		C,0.0265410998,7.2313908609,0.0020061288
		H,−0.00238608,8.3086192848,0.0024098658
		C,0.0002095158,0.0001128112,−1.0186816208
		B,−0.9007934185,1.239906048,−0.2388091951
p-$B_{10}H_{12}C_2$:NCH (HB)	−424.15127447	B,−1.457545477,−0.4735828608,−0.2391488039
		B,−1.4561994292,0.4728866085,1.2714880294
		C,−0.0004798181,−0.0002396106,2.0530023808
		B,1.4555973794,0.4728996962,1.272134252

		B,0.8994783584,−1.238617939,1.2718227843
		B,1.4576009399,−0.4735788993,−0.2384869618
		B,0.0000376683,−1.5325599646,−0.2389473669
		B,−0.0002947518,1.5306677174,1.2719312765
		H,−0.0004463461,2.5379160901,1.9081892205
		H,0.000187182,−2.5374032133,−0.8793105518
		H,−0.0007576011,−0.0003921352,3.1457801432
		H,−2.4143626063,0.784082244,1.907437221
		H,2.4134709693,−0.7840331634,−0.8785419245
		H,1.4914514832,−2.0536575892,1.9079813468
		H,−2.4131279894,−0.7840490904,−0.8796415473
		H,2.41348459,0.7841008926,1.9085153543
		H,−1.4913273165,2.0529866075,−0.8790892008
		B,−0.9001035433,−1.2386151477,1.2714126847
		B,0.9008601859,1.2399107056,−0.2384022809
		H,−1.4923331844,−2.0536290829,1.9073111768
		H,1.4916744184,2.0529725862,−0.8784051171
		H,0.0004601632,0.000243605,−2.111317005
		N,0.0004995598,0.0003188632,5.4005557988
		C,0.0011006206,0.0006274828,6.58206864
		H,0.0016584473,0.0007267383,7.6595335606
		C,0.0005201514,0.0000011389,−0.9959567457
		B,−0.9026539807,1.2425172725,−0.2217833214
		B,−1.4605798214,−0.4746048532,−0.2221030514
		B,−1.4562007843,0.4728912121,1.2860563046
		C,−0.0012021085,−0.00000105,2.0691357887
		B,1.4546755528,0.4728936713,1.2876742052
		B,0.8987441126,−1.2380583665,1.2873721898
		B,1.4607543111,−0.4745939293,−0.2204581916
		B,0.0000837666,−1.5358312205,−0.2212801862
		B,−0.0007507305,1.5303242847,1.2868682371
		H,−0.0010984009,2.5395253277,1.9189818138
		H,0.0004510285,−2.5245480744,−0.8841049762
		H,−0.0017690792,−0.0000334932,3.1617040793
p-$B_{10}H_{11}C_2$-1F:NCH (HB)	−523.19391774	H,−2.4163691072,0.7847538907,1.9176140821
		H,2.4014484451,−0.7801308533,−0.8827608278
		H,1.4915808737,−2.0545251447,1.9198042505
		H,−2.4005283678,−0.7801281927,−0.8854641597
		H,2.4141199166,0.7847488963,1.9203270611
		H,−1.4834282306,2.0424124489,−0.8849334898
		B,−0.9002694131,−1.2380693606,1.2863612968
		B,0.9028295314,1.2425173225,−0.2207754581
		H,−1.4938168749,−2.0545468921,1.9181217866
		H,1.4843522272,2.0424054631,−0.8832693001
		F,0.0012826771,0.0000045597,−2.3680950498
		N,0.0010778873,0.0000339818,5.40362567
		C,0.002578585,−0.0000068494,6.5850936927
		H,0.0041678326,0.0000488098,7.6626265934
p-$B_{10}H_{11}C_2$-1Cl:NCH (HB)	−883.20942082	C,0.0000077556,−0.0002550938,−0.9900636968

		B,−0.9031859244,1.2430899952,−0.2075318463
		B,−1.4613865385,−0.4748668117,−0.2070673322
		B,−1.4550885671,0.4731670143,1.3016405301
		C,−0.0000363834,0.0006122421,2.0844779952
		B,1.4550415023,0.473179658,1.3016729593
		B,0.8992453982,−1.2373536502,1.3021640949
		B,1.461380234,−0.4748585849,−0.2070006008
		B,−0.0000048457,−1.5366119802,−0.2067348915
		B,−0.0000171393,1.5303399322,1.3013566466
		H,−0.000033963,2.5407040672,1.9320654422
		H,0.0000120002,−2.528872473,−0.8641743793
		H,0.0000078131,0.0008429857,3.1772707836
		H,−2.4158528284,0.7855166806,1.9325147106
		H,2.4049060416,−0.7816231881,−0.864631625
		H,1.49302108,−2.05445064,1.9333582879
		H,−2.4048884302,−0.7816274807,−0.8647236267
		H,2.4157921504,0.7855234221,1.9325808577
		H,−1.4862922036,2.0455079362,−0.8654972968
		B,−0.8993138241,−1.2373604123,1.302126209
		B,0.9031759287,1.2430902945,−0.207511952
		H,−1.4931036191,−2.0544505557,1.9333222478
		H,1.486303852,2.0455110784,−0.8654570377
		Cl,0.0000408932,−0.0007553978,−2.7577217327
		N,0.0000417368,−0.0005482369,5.4178772656
		C,0.000083075,−0.0013701898,6.5992750156
		H,0.0001448058,−0.0020806114,7.6767952656
		C,0.0001817779,0.0002198809,−0.8630032971
		B,−0.9024284032,1.2424004158,−0.0761327554
		B,−1.4602714831,−0.4743814449,−0.0763550297
		B,−1.4567298678,0.4733195949,1.4321417026
		C,−0.0001062127,−0.0000710725,2.2137095911
		B,1.4566766624,0.4732937349,1.4324385949
		B,0.900247151,−1.2391498936,1.4322194397
		B,1.4604886247,−0.4743813506,−0.0760644102
		B,0.000116357,−1.5353740278,−0.0763150799
		B,−0.0000028558,1.5316572638,1.4323798383
		H,−0.0000629279,2.5395099991,2.0672253891
p-$B_{10}H_{11}C_2$-1Cl:NCH (XB)	−883.20517392	H,0.0001817236,−2.5296962687,−0.7301926378
		H,−0.0002174005,−0.0001771564,3.3060250825
		H,−2.4153934057,0.7847480457,2.0668441019
		H,2.4062898018,−0.7816182766,−0.7298035743
		H,1.492624031,−2.0546287906,2.0669543698
		H,−2.4059520407,−0.7815961152,−0.7302728398
		H,2.4152080065,0.7846944297,2.0673278503
		H,−1.4868414958,2.0469670081,−0.7298949447
		B,−0.9003249924,−1.2391386629,1.4320314455
		B,0.9026927289,1.2423827448,−0.0759697513
		H,−1.4928270951,−2.0546223969,2.066659699
		H,1.487257077,2.0469285169,−0.7296317116
		Cl,0.0003741553,0.0004195716,−2.6271345421

		N,−0.0002006394,−0.0004257421,−5.6775363205<br>C,−0.0003888851,−0.0006355035,−6.8600498475<br>H,−0.0005903821,−0.000644504,−7.9372179379
p-$B_{10}H_{11}C_2$-1Br:NCH (HB)	−2996.09134178	C,0.0000431711,−0.0002980943,−0.9831602998<br>B,−0.9032756744,1.2432422072,−0.2028169834<br>B,−1.4615644438,−0.4749514815,−0.2022953364<br>B,−1.4551810198,0.4732302125,1.3068797742<br>C,−0.0000742852,0.0006921232,2.0895851874<br>B,1.4550962803,0.4732591994,1.3069618911<br>B,0.8992907056,−1.2373685489,1.3074917747<br>B,1.461564194,−0.4749392942,−0.2021906908<br>B,0.000011224,−1.5368545624,−0.2018908413<br>B,−0.0000430784,1.5304635603,1.306587988<br>H,−0.0000832101,2.5411017878,1.9370245874<br>H,0.0000362524,−2.5313527168,−0.8562394255<br>H,−0.0000758429,0.0009880766,3.1824292762<br>H,−2.4162069234,0.7856880389,1.9374552965<br>H,2.4072206627,−0.7823913994,−0.8567281667<br>H,1.493195507,−2.054612448,1.9385531533<br>H,−2.4071744653,−0.7824003624,−0.8569058599<br>H,2.4160588909,0.7856988725,1.9376570171<br>H,−1.4876928143,2.0474183936,−0.8577666092<br>B,−0.8993585893,−1.2373843974,1.3074634468<br>B,0.9033118951,1.24326678,−0.202748924<br>H,−1.493309972,−2.0546527292,1.9384485963<br>H,1.4877522618,2.047457021,−0.8576687245<br>Br,0.0001050328,−0.0009071528,−2.9028316536<br>N,0.0000282814,−0.0005053036,5.422254555<br>C,0.0001405736,−0.0014937028,6.6036544129<br>H,0.0001853861,−0.0023940791,7.6811788209
p-$B_{10}H_{11}C_2$-1Br:NCH (XB)	−2996.08909232	C,0.0000032275,0.0000082533,−0.8428913305<br>B,−0.9024991613,1.2420332435,−0.0578037312<br>B,−1.4601230378,−0.4744934225,−0.0577098812<br>B,−1.4567737823,0.4733429253,1.4513702597<br>C,0.0000192871,0.0001759871,2.232770861<br>B,1.4567423834,0.4736005844,1.4514112021<br>B,0.9004475046,−1.2390005317,1.4514972187<br>B,1.4601850013,−0.4742499127,−0.0577079471<br>B,0.0001425831,−1.535203689,−0.0576389556<br>B,−0.000110026,1.5318602128,1.4513254382<br>H,−0.0002298596,2.5400076107,2.0859368579<br>H,0.0002346592,−2.5322586267,−0.7079579699<br>H,0.000001468,0.0002402689,3.3251964324<br>H,−2.4155696815,0.7848418649,2.0860100193<br>H,2.4084306814,−0.7823281842,−0.7080695442<br>H,1.4930703399,−2.0544999834,2.0862040187<br>H,−2.4083111081,−0.7827013176,−0.7081026885<br>H,2.4154824219,0.7852330925,2.086061036<br>H,−1.4885888842,2.0485710676,−0.7082276897

		B,−0.9002073023,−1.239137979,1.4514733011
		B,0.9023105839,1.2422002622,−0.0577891943
		H,−1.4926754099,−2.0547433092,2.0861858313
		H,1.4882812648,2.0488157681,−0.7082174078
		Br,0.0000430694,−0.0001011582,−2.7612209809
		N,−0.0001092262,−0.0005829033,−5.7833974895
		C,−0.0000998437,−0.000738143,−6.9655281886
		H,−0.0000971599,−0.0008919807,−8.0427970098
		C,0.0001622558,−0.0006655717,−0.9778812878
		B,−0.9031783217,1.2431151471,−0.1968477183
		B,−1.4614909194,−0.4749240145,−0.1956877091
		B,−1.4554903489,0.4739324066,1.3138914363
		C,−0.0004054442,0.0016434839,2.0967275566
		B,1.455046775,0.4738765485,1.314353145
		B,0.8991139624,−1.2368990363,1.3155696611
		B,1.4615515389,−0.4749651438,−0.1951436148
		B,−0.0000080579,−1.536788156,−0.194558469
		B,−0.0001595747,1.5311632732,1.3132944787
		H,−0.0003051338,2.5422834873,1.9431741841
		H,0.0000725335,−2.5354092423,−0.8432536257
		H,−0.0005681226,0.0023755506,3.1895952876
p-$B_{10}H_{11}C_2$-1I:NCH (HB)	−718.34943470	H,−2.4168043191,0.7867860992,1.9440330929
		H,2.410953908,−0.7839551001,−0.8441887867
		H,1.4930298871,−2.0540585988,1.9468550031
		H,−2.4106486236,−0.7838662737,−0.8450863062
		H,2.4160980909,0.7866084248,1.9449462216
		H,−1.4897477232,2.0501332719,−0.8469918593
		B,−0.8997367674,−1.2368454197,1.3152667457
		B,0.9033432421,1.2432179497,−0.1965451306
		H,−1.4939097151,−2.0540059193,1.9463689944
		H,1.490144507,2.050218771,−0.8464995236
		I,0.0005174705,−0.0022938596,−3.1096779692
		N,0.0002538318,−0.0014078433,5.4294172077
		C,0.0007524133,−0.00366902,6.6108525551
		H,0.0014126552,−0.0056012146,7.6883986932
		C,−0.0002198849,−0.0001971865,−0.8092056817
		B,−0.9018861363,1.2416129434,−0.0216388424
		B,−1.4597133207,−0.473987838,−0.0212891855
		B,−1.4567060935,0.4738467048,1.4886096886
		C,0.0001227068,0.0003400039,2.2697443944
		B,1.4569790556,0.4732651078,1.488329821
p-$B_{10}H_{11}C_2$-1I:NCH (XB)	−718.34920760	B,0.900161815,−1.2392536676,1.4886730201
		B,1.4592401084,−0.4745783865,−0.0215770694
		B,−0.0004349908,−1.5346401358,−0.0212594497
		B,0.0003394938,1.5320222617,1.4882776427
		H,0.0005943956,2.5402714858,2.1229487866
		H,−0.0006951841,−2.5361336178,−0.6655262452
		H,0.0002309638,0.0005278989,3.3623059304
		H,−2.4153783901,0.785705735,2.1234948896

	H,2.4114839546,−0.7843166248,−0.666058864<br>H,1.4926430459,−2.0548919338,2.1235953425<br>H,−2.412231113,−0.7833653092,−0.6655672913<br>H,2.4159189312,0.7847121215,2.123032363<br>H,−1.4904103959,2.0517708785,−0.6661689153<br>B,−0.9005878175,−1.2388814798,1.4888495716<br>B,0.902124329,1.2412480023,−0.0218157137<br>H,−1.4932725873,−2.0542772334,2.1238971578<br>H,1.4908323548,2.0511690621,−0.6664745289<br>I,−0.0003957429,−0.0005444473,−2.9425527414<br>N,0.0002085483,−0.0005240421,−6.013799653<br>C,0.000437651,−0.0004855667,−7.1954396618<br>H,0.000614296,−0.0004147362,−8.2730022974

Table S3. ρ_{BCP}, $\nabla 2\rho_{BCP}$ and H_{BCP} (au) of the intermolecular BCPs in the 1-halo-*closo*-carboranes:NCH complexes.

	Atoms	ρ_{BCP}	$\nabla 2\rho_{BCP}$	H_{BCP}
o−$B_{10}H_{12}C_2$:NCH (HB)	N25−H24	0.0103	0.0336	0.0009
	H19−N25	0.0103	0.0335	0.0009
o−$B_{10}H_{11}C_2$−1F:NCH (HB)	N25−H19	0.0174	0.0534	0.0011
o−$B_{10}H_{11}C_2$−1Cl:NCH (HB)	N25−H19	0.0177	0.0543	0.0011
o−$B_{10}H_{11}C_2$−1Br:NCH (HB)	H19−N25	0.0178	0.0548	0.0011
o−$B_{10}H_{11}C_2$−1I:NCH (HB)	H19−N25	0.0178	0.0548	0.0011
m−$B_{10}H_{11}C_2$:NCH (HB)	N25−H23	0.0151	0.0458	0.0009
m−$B_{10}H_{11}C_2$−1F:NCH (HB)	H23−N25	0.0157	0.0476	0.0009
m−$B_{10}H_{11}C_2$−1Cl:NCH (HB)	N25−H23	0.0157	0.0477	0.0009
m−$B_{10}H_{11}C_2$−1Br:NCH (HB)	N25−H23	0.0157	0.0477	0.0009
m−$B_{10}H_{11}C_2$−1I:NCH (HB)	N25−H23	0.0156	0.0475	0.0009
p−$B_{10}H_{11}C_2$:NCH (HB)	H13−N25	0.0147	0.0443	0.0008
p−$B_{10}H_{11}C_2$−1F:NCH (HB)	H13−N25	0.0150	0.0455	0.0009
p−$B_{10}H_{11}C_2$−1Cl:NCH (HB)	N25−H13	0.0151	0.0456	0.0009
p−$B_{10}H_{11}C_2$−1Br:NCH (HB)	N25−H13	0.0151	0.0457	0.0009
p−$B_{10}H_{11}C_2$−1I:NCH (HB)	N25−H13	0.0151	0.0457	0.0009
o−$B_{10}H_{11}C_2$−1Cl:NCH (XB)	N25−Cl24	0.0107	0.0406	0.0016
m−$B_{10}H_{11}C_2$−1Cl:NCH (XB)	Cl24−N25	0.0101	0.0380	0.0015
p−$B_{10}H_{11}C_2$−1Cl:NCH (XB)	Cl24−N25	0.0099	0.0369	0.0015
o−$B_{10}H_{11}C_2$−1Br:NCH (XB)	N25−Br24	0.0133	0.0472	0.0016
m−$B_{10}H_{11}C_2$−1Br:NCH (XB)	N25−Br24	0.0126	0.0444	0.0016
p−$B_{10}H_{11}C_2$−1Br:NCH (XB)	N25−Br24	0.0123	0.0435	0.0015
o−$B_{10}H_{11}C_2$−1I:NCH (XB)	I24−N25	0.0156	0.0493	0.0011
m−$B_{10}H_{11}C_2$−1I:NCH (XB)	I24−N25	0.0145	0.0459	0.0011
p−$B_{10}H_{11}C_2$−1I:NCH (XB)	I24−N25	0.0142	0.0449	0.0011

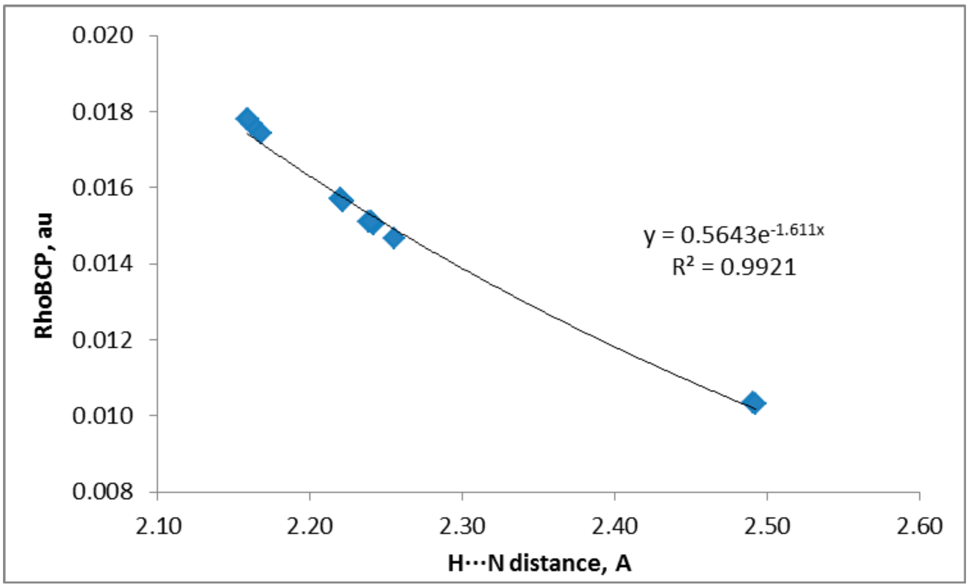

Figure S1. ρBCP (au) vs. the interatomic N···H distance (Å) in the 1-halo-*closo*-carboranes:NCH (HB) complexes.

Table S4. NEDA partition terms, kJ mol^{-1}, of the 1-halo-*closo*-carboranes:NCH complexes.

	**Charge Transfer**	**Electrostatic**	**Polarization**	**Exchange Correlation**	**DEF 1–halcarborane**	**DEF NCH**
o–$B_{10}H_{12}C_2$:NCH (HB)	−39.6	−23.3	−34.8	−23.9	29.0	77.9
o–$B_{10}H_{11}C_2$–1F:NCH (HB)	−57.3	−23.5	−29.2	−16.9	22.0	91.1
o–$B_{10}H_{11}C_2$–1Cl:NCH (HB)	−59.1	−23.3	−30.5	−18.0	25.3	92.9
o–$B_{10}H_{11}C_2$–1Br:NCH (HB)	−59.8	−23.2	−31.5	−18.5	26.5	94.0
o–$B_{10}H_{11}C_2$–1I:NCH (HB)	−59.8	−23.1	−32.7	−19.2	28.4	94.3
m–$B_{10}H_{12}C_2$:NCH (HB)	−46.6	−17.7	−23.2	−14.4	17.7	74.7
m–$B_{10}H_{11}C_2$–1F:NCH (HB)	−48.8	−19.6	−24.4	−14.9	18.4	78.1
m–$B_{10}H_{11}C_2$–1Cl:NCH (HB)	−48.9	−19.5	−24.5	−14.9	18.4	78.4
m–$B_{10}H_{11}C_2$–1Br:NCH (HB)	−48.9	−19.5	−24.5	−14.9	18.5	78.4
m–$B_{10}H_{11}C_2$–1I:NCH (HB)	−48.7	−19.2	−24.3	−14.8	18.3	78.0
p–B10H12C2:NCH (HB)	−45.6	−16.2	−22.6	−14.1	17.2	72.9
p–$B_{10}H_{11}C_2$–1F:NCH (HB)	−46.9	−17.8	−23.5	−14.4	17.5	75.3
p–$B_{10}H_{11}C_2$–1Cl:NCH (HB)	−47.0	−17.8	−23.5	−14.4	17.6	75.4
p–$B_{10}H_{11}C_2$–1Br:NCH (HB)	−47.1	−17.8	−23.6	−14.4	17.6	75.6
p–$B_{10}H_{11}C_2$–1I:NCH (HB)	−47.1	−17.6	−23.5	−14.4	17.6	75.5
o–$B_{10}H_{11}C_2$–1Cl:NCH (XB)	−17.8	−11.4	−49.7	−16.4	39.2	50.9
o–$B_{10}H_{11}C_2$–1Br:NCH (XB)	−28.2	−18.3	−81.7	−22.8	56.2	86.2
o–$B_{10}H_{11}C_2$–1I:NCH (XB)	−44.0	−27.1	−64.3	−27.8	75.4	73.0
m–$B_{10}H_{11}C_2$–1Cl:NCH (XB)	−16.2	−8.1	−44.7	−15.2	36.4	45.3
m–$B_{10}H_{11}C_2$–1Br:NCH (XB)	−25.6	−14.6	−74.5	−21.3	52.5	78.0
m–$B_{10}H_{11}C_2$–1I:NCH (XB)	−40.0	−22.6	−58.0	−25.7	69.2	65.8
p–$B_{10}H_{11}C_2$–1Cl:NCH (XB)	−15.6	−7.0	−43.3	−14.8	35.2	43.8
p–$B_{10}H_{11}C_2$–1Br:NCH (XB)	−24.9	−13.5	−72.8	−20.8	51.3	76.1
p–$B_{10}H_{11}C_2$–1I:NCH (XB)	−39.0	−21.2	−56.6	−25.2	67.5	64.2

Table S5. CH···N distances (Å) in the CSD search between carboranes and N-bases.

o−carboranes		m−carboranes		p−carboranes	
**Refcode**	**CH···N**	**Refcode**	**CH···N**	**Refcode**	**CH···N**
MOYHEQ	2.394	DOVXOE	2.814	IDIHOT	2.404
MOYHEQ	2.717	DOVXOE	2.649	IMEQIB	2.79
MOYHEQ	2.111	DOVYIZ	2.793	JABGIF	2.603
VOZZAO	2.507	DOVYIZ	2.61	JABGIF	2.331
XUHZEI	2.164	DOVYOF	2.363	MIFJUH	2.82
BOKKOB	2.533	DOVYOF	2.713	MIFJUH	2.455
BOVQUY	2.891	DOWMIO	2.389	MIFKES	2.76
BOVQUY	2.279	FEZJUR	2.281	MIFKES	2.79
BOVQUY	2.502	FEZJUR	2.373		
BOZXAR	2.6	FEZJUR	2.915		
BOZXAR	2.864	FEZJUR	2.863		
BUQXEQ	2.816	GISFUM	2.456		
COXPEM	2.492	GISGAT	2.841		
COXPEM	2.603	GISGAT	2.933		
ECIRIS	2.905	IDIGUY	2.442		
GAFFEB	2.683	IDIGUY	2.327		
GAFFEB	2.986	IMEMAQ	2.472		
GAFFEB	2.856	IMEMAQ	2.51		
GAFFEB	2.732	IMEMAQ	2.891		
HIYGEE	2.218	IZAHUN	2.616		
ICUSAD	2.907	IZAJAV	2.156		
JABGOL	2.887	IZAJAV	2.295		
JABGOL	2.48	MATLAX	2.514		
LAQGIW	2.315	OWITEV	2.253		
LUTGAI	2.526	OWITEV	2.765		
NACGIH	2.498	OWITOF	2.218		
NACGIH01	2.498	OWITOF	2.188		
NEQXOZ	2.246	QUKHOU	2.663		
NEQXOZ	2.776	QUKHUA	2.672		
NEQXOZ	2.816	RECYUV	2.709		
NEQXOZ	2.754	TAVXIY	2.99		
NEQXOZ	2.448	TIWCIO	2.648		
NURLER	2.468	TIWCUA	2.374		
PECHEM	2.69	TIWCUA	2.419		
POVVAZ	2.601	TOKMAK	2.721		
POVVAZ	2.278	TOKMAK	2.575		
QUKHIO	2.276	TOKMEO	2.65		
RASYIV	2.891	TOKMEO	2.935		
RASYIV	2.447	TOKMIS	2.766		
REJMUO01	2.402	TOKMOY	2.71		
RINQUD	2.988	TOKMUE	2.51		
ROHWAM	2.865	TOKNAL	2.472		
TELLAX	2.844	TOKNAL	2.535		
TULQIC	2.954	UDOSUE	2.546		
VEPVET	2.462	UDOSUE	2.613		
VEPVET	2.804	VEPVIX	2.955		
WADPID	2.388	VEPVIX	2.68		

XARCIC	2.517	VEPVIX	2.766
XARCIC	2.617	VEPVIX	2.493
YADJAP	2.721	YIZXUB	2.413
YADJET	2.775	YIZYAI	2.334
YADJET	2.836	YIZYIQ	2.868
		YIZYOW	2.848
